# Growth arrest‐specific protein 2 (GAS2) interacts with CXCR4 to promote T‐cell leukemogenesis partially via c‐MYC

**DOI:** 10.1002/1878-0261.13306

**Published:** 2022-09-11

**Authors:** Wenjuan Ma, Yan Wan, Jianxiang Zhang, Jianan Yao, Yifei Wang, Jinchang Lu, Hong Liu, Xiaorui Huang, Xiuyan Zhang, Haixia Zhou, Yulong He, Depei Wu, Jianrong Wang, Yun Zhao

**Affiliations:** ^1^ Cyrus Tang Medical Institute, Collaborative Innovation Center of Hematology Soochow University Suzhou China; ^2^ Key Laboratory of Thrombosis and Hemostasis, Ministry of Health The First Affiliated Hospital of Soochow University Suzhou China; ^3^ National Clinical Research Center for Hematologic Diseases Suzhou China; ^4^ Cam‐Su Genomic Resources Center Soochow University Suzhou China; ^5^ State Key Laboratory of Radiation Medicine and Radioprotection Soochow University Suzhou China; ^6^ MOE Engineering Center of Hematological Disease Soochow University Suzhou China; ^7^ Key Laboratory of Stem Cells and Biomedical Materials of Jiangsu Province and Chinese Ministry of Science and Technology Suzhou China

**Keywords:** c‐MYC, CXCR4, GAS2, hematopoiesis, leukemogenesis, T‐ALL

## Abstract

Although growth arrest‐specific protein 2 (GAS2) promotes the growth of T‐cell acute lymphoblastic leukemia (T‐ALL) cells in culture, the effect of GAS2 on T‐cell leukemogenesis has not been studied, and the mechanism remains unclear. In the present study, xenograft studies showed that *GAS2* silencing impaired T‐cell leukemogenesis and decreased leukemic cell infiltration. Mechanistically, GAS2 regulated the protein expression of C‐X‐C chemokine receptor type 4 (CXCR4) rather than its transcript expression. Immunoprecipitation revealed that GAS2 interacted with CXCR4, and confocal analysis showed that GAS2 was partially co‐expressed with CXCR4, which provided a strong molecular basis for GAS2 to regulate CXCR4 expression. Importantly, *CXCR4* overexpression alleviated the inhibitory effect of *GAS2* silencing on the growth and migration of T‐ALL cells. Moreover, *GAS2* or *CXCR4* silencing inhibited the expression of *NOTCH1* and *c‐MYC*. Forced expression of *c‐MYC* rescued the growth suppression induced by *GAS2* or *CXCR4* silencing. Meanwhile, *GAS2* deficiency, specifically in blood cells, had a mild effect on normal hematopoiesis, including T‐cell development, and *GAS2* silencing did not affect the growth of normal human CD3^+^ or CD34^+^ cells. Overall, our data indicate that GAS2 promotes T‐cell leukemogenesis through its interaction with CXCR4 to activate NOTCH1/c‐MYC, whereas impaired *GAS2* expression has a mild effect on normal hematopoiesis. Therefore, our study suggests that targeting the GAS2/CXCR4 axis is a potential therapeutic strategy for T‐ALL.

## Graphical abstract

Growth arrest‐specific (GAS2) interacts with C‐X‐C motif chemokine receptor 4 (CXCR4) and enhances the stability of CXCR4 in T‐cell acute lymphoblastic leukemia (T‐ALL) cells, and the GAS2/CXCR4 axis activates the expression of NOTCH/c‐MYC and promotes T‐cell leukemogenesis. Therefore, our study suggests that targeting the GAS2/CXCR4 axis is a potential therapeutic strategy for T‐ALL.

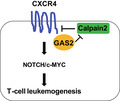

**Abbreviations**
CFC: colony‐forming cell
CXCL12: C‐X‐C motif chemokine ligand 12
CXCR4: C‐X‐C motif chemokine receptor type 4
EPO: erythropoietin.
GAS2: growth arrest‐specific protein 2
GAS2DN: dominant negative form of GAS2
G‐CSF: granulocyte colony‐stimulating factor
GM‐CSF: granulocyte–macrophage colony‐stimulating factor
IL‐2: interleukin‐2
IL‐3: interleukin‐3
IL‐6: interleukin‐6
IP: immunoprecipitation
NBM: normal bone marrow
RT‐qPCR: reverse transcription‐quantitative polymerase chain reaction
SCF: stem cell factor
T‐ALL: T‐cell acute lymphoblastic leukemia

## 1 Introduction

Growth arrest‐specific protein 2 (GAS2) is a component of microfilaments that plays an important role in many cellular processes, such as cytoskeletal regulation, cell cycle, apoptosis and senescence [1, 2, 3, 4, 5]. Recently, GAS2 mutations in both humans and mice have been found to lead to hearing loss due to the destabilization of microtubule bundles in inner ear supporting cells [6]. In addition, GAS2 is an endogenous inhibitor of Clapain2 (a calcium‐dependent protease) [7]; the truncated GAS2 (GAS2ΔA71–313) can bind Calpain2 but not to inhibit its protease activity, and is thus termed the dominant negative form of GAS2 (GAS2DN). Calpain plays a crucial role in cancer development. For example, deregulated expression or activity of Calpain has been reported in various cancers, and many compounds with proven anticancer efficacy can modulate Calpain activities [8]. Accumulating evidence has shown that the GAS2/Calpain2 axis plays a dual role in cancer cells [1]. On one hand, GAS2/Calpain2 has been shown to stabilize p53 and sensitize cancer cells upon treatment of etoposide [7], or to prevent the malignant transformation of normal cells by promoting cellular senescence [5]. In contrast, GAS2/Calpain2 plays an oncogenic role in some other malignant cells partially through its regulation of β‐catenin [9, 10, 11, 12, 13, 14, 15, 16], including T‐cell acute lymphoblastic leukemia (T‐ALL) cells [13]. However, the effect of GAS2 on the *in vivo* growth of T‐ALL cells has not been studied, and the functional mechanisms of GAS2 have not been fully defined in these cells.

T‐ALL is a fatal hematological malignancy that accounts for 15% and 25% of pediatric and adult ALL cases, respectively, and is prone to early relapse [17, 18]. Although the outcome of T‐ALL has improved especially in children, the survival of patients with relapse remains dismal [17, 18, 19, 20]. Many reports have demonstrated the importance of the NOTCH/c‐MYC pathway and CXCR4 signaling in T‐ALL pathogenesis [21, 22, 23, 24, 25, 26]. Importantly, inhibition of either NOTCH or CXCR4 signaling holds promise for improving the management of T‐ALL [24, 27, 28, 29, 30]. Nevertheless, whether the GAS2/Calpain2 axis has any connection with either NOTCH1/c‐MYC or CXCR4 signaling remains unclear.

Our previous work has shown that the dominant negative form of GAS2 (GAS2DN) exhibits a stronger inhibitory effect on chronic myeloid leukemia (CML) CD34^+^ cells than on normal CD34^+^ cells [12]; however, the effect of Gas2 impairment on normal hematopoietic cells has not been studied yet [31], which is critical to determine whether targeting GAS2 is a proper approach to combat hematological malignancies.

In this study, our results demonstrated that GAS2 interacts with CXCR4 to promote T‐cell leukemogenesis, and the GAS2/CXCR4 axis sustains the growth of T‐ALL cells partially via the activation of NOTCH1/c‐MYC signaling. In contrast, GAS2 impairment had mild effects on normal hematopoietic cells. In summary, our study demonstrated that GAS2 plays a critical role in the growth of T‐ALL cells and suggests that GAS2 is a novel therapeutic target for this disease.

## 2 Materials and methods

### 2.1 Patients and cells

Jurkat cells were obtained from the Cell Bank of the Chinese Academy of Sciences (Shanghai, China), free of mycoplasma contamination, and confirmed by authentication tests. Bone marrow cells of T‐ALL patients and healthy donors were from the Hematological Biobank, Jiangsu Biobank of Clinical Resources, and informed consent forms were approved by the Ethics Committee of Soochow University (ECSU‐2019000125, Suzhou, China), in accordance with the Declaration of Helsinki. The experiments were undertaken with the understanding and written consent of each subject. The clinical characteristics of T‐ALL patients are summarized (Table S1). Nucleated cells were obtained using a gradient centrifuge with Lympholyte‐H cell separation media (Cedarlane Laboratories, Burlington, NC, USA), and CD3^+^ or CD34^+^ cells were enriched using an EasySep kit (STEMCELL Technologies, Vancouver, BC, Canada).

### 2.2 RNA extraction and RT‐qPCR

RNA preparation and gene expression analyses were performed as previously described [12]. The sequences of gene‐specific primers are summarized (Table S2). For RT‐qPCR analysis, the expression of each individual transcript was normalized to that of *β‐ACTIN*. To compare the expression of individual transcripts in different samples, the expression in the test group was normalized to that in the control group and was shown as relative expression.

### 2.3 DNA methylation analysis

The DNA methylation content of *GAS2* promoter region was analyzed as previously described [32]. T‐ALL samples or normal CD3^+^ samples were treated with bisulfate, and each bisulfate‐treated sample was amplified by PCR using the following primers: forward, 5′‐AATTTGTGGGGATTAGTATATTTAG‐3′; and reverse, 5’‐AATATCAAAAACAATTATCTCCAAC‐3′. The PCR product of each sample was subcloned into a T‐vector, and several clones (typically 4–6) were sequenced to estimate the DNA methylation content.

### 2.4 Western blot and immunoprecipitation

Protein samples were prepared and western blot was performed as previously described [12]. For the immunoprecipitation (IP) assay, the protein lysate (500 μg) was incubated with anti‐GAS2 or anti‐CXCR4 antibodies, and same amount of protein lysate was incubated with the appropriate isotype control antibody. The mixture was then incubated with protein G Plus‐Agarose (L‐00209; GenScript, Piscataway, NJ, USA). Lastly, the precipitates were analyzed by immunoblot. In coimmunoprecipitation (Co‐IP) assay, Flag‐GAS2 and/or HA‐CXCR4 were overexpressed in 293 T cells, and then the protein lysate were subjected to immunoblot and IP assays. The antibody information is listed (Table S3).

### 2.5 Lentiviral vectors, viral production, and transduction

Lentiviral vector that overexpresses GAS2DN has been constructed previously [12]. CXCR4 cDNA was subcloned into a lentiviral vector using the following primers: forward, 5′‐AATCTAGAATGTCCATTCCTTTGCCTCTTTTGCA‐3′ (*Xba*I site is underlined); and reverse, 5′‐AACATATGTTAGCTGGAGTGAAAACTTGAAGACT‐3′ (*Nde*I site is underlined). c‐MYC cDNA was subcloned into a lentiviral vector using the following primers: forward, 5′‐AATCTAGACTGGATTTTTTTCGGGTAGTGG (*Xba*I site is underlined); and reverse, AACATATGTTACGCACAAGAGTTCCGTAGC‐3′ (*Nde*I site is underlined). Lentiviral vectors to silence GAS2, CXCR4, Calpain2, and the scrambled control were obtained from GenePharma Co., Ltd. (Shanghai, China), the sequences of these short hairpin RNAs (shRNAs) are listed (Table S4). Lentiviral production was performed as previously described [12].

Normal CD3^+^ cells and T‐ALL cells from patients were cultured with ImmunoCult‐XF T cell expansion medium (#10981, STEMCELL Technologies) supplemented with IL‐2 (2 ng·mL^−1^, #78036, STEMCELL Technologies), and then activated with CD3/CD28 antibodies (B281555 and B284044, Biolegend, San Diego, CA, USA). Transduced cells (GFP^+^) were purified using fluorescence‐activated cell sorting (FACS) (BD FACSAria III, Becton Dickinson, Franklin Lakes, NJ, USA). CD34^+^ cells were transduced as previously described [12].

Throughout the study, transduced cells were all sorted based on the expression of fluorescent proteins, and their cellular and molecular properties were analyzed.

### 2.6 Animals

Female immunodeficient mice 6–8 weeks of age (NOD.CB17‐*Prkdc* ^*scid*^ *Il2rg* ^*tm1*^/Bcgen, Biocytogen, Beijing, China) were maintained in the specific pathogen free (SPF) animal facility of Soochow University. Based on the weight the mice were randomly allocated to each group (4 mice or less/cage), and leukemic cells were injected into the mice through tail vein. These mice were monitored for signs of weight loss or lethargy, twice a week in the first 3 weeks post injection and every day afterwards. They were euthanized in CO_2_ chamber when manifesting disease symptoms or becoming moribund. The mice were then dissected, and cells from the spleen, bone marrow, and peripheral blood were analyzed by flow cytometry (Gallios, Beckman Coulter, Brea, CA, USA).

The exon3 of *Gas2* was flanked by flox to generate Gas2^flox/flox^ mice (Biocytogen). Gas2^flox/flox^ mice were crossed with Vav‐iCre mice to obtain flox/flox;Vav‐iCre mice. Tail genomic DNA was obtained for genotyping using specific primers (Table S5). The hematopoietic cells of Gas2^flox/flox^ mice and flox/flox;Vav‐iCre mice were analyzed by flow cytometry and the coefficients (ratio of organ weight to body weight) of various organs of these two groups of mice were measured and compared.

All studies were conducted following an institutional protocol approved by the Ethics Committee of Soochow University (ECSU‐2019000124, Suzhou, China).

### 2.7 Flow cytometry analysis

Cells from the bone marrow, spleen, and peripheral blood of the mice were harvested in 2% (vol/vol) fetal bovine serum‐supplemented Hank's Balanced Salt Solution (collectively called HF). The cells were blocked with 2% HF in addition with CD16/32 (223142, Becton Dickinson), and stained with various antibodies from eBioscience/Thermo Fisher Scientific, Waltham, MA, USA, including anti‐Ter‐119 (17‐5921‐82), anti‐Gr‐1 (45‐5931‐80), anti‐Mac‐1 (11‐0112‐82), anti‐B220 (25‐0452‐82), anti‐CD4 (11‐0041‐82), and anti‐CD8 (12‐0081‐82), for flow cytometry analysis. Jurkat cells were first blocked with 5% human serum in 2% HF and then stained with anti‐CD184 (555 974, Becton Dickinson) for flow cytometry analysis.

### 2.8 Transwell assay

The cells were starved with RPMI‐1640 medium supplemented with 0.5% bovine serum albumin (BSA) for 4 h, 10 000 cells were then transferred to the upper chamber of each well in a transwell plate, and the lower chamber was supplied with the same medium plus CXCL12 (101 492, Proteintech, Rosemont, IL, USA). After incubation at 37 °C for 2 h, the cells in the lower chamber were counted, the number of cells in lower chamber of the test group was normalized to that of the control group to calculate the relative migration of the test cells.

### 2.9 Immunofluorescence assay

Jurkat cells were processed for an immunofluorescence assay as previously described [13]. Briefly, the cells were first incubated with antibody against GAS2 (ab109762; Abcam, Waltham, MA, USA) or Calpain2 (A4066; ABclonal, Wuhan, China), and they were incubated with antibody against CXCR4 (60042‐1‐Ig; Proteintech). The cells were then incubated with FITC‐conjugated anti‐rabbit IgG (GAR001; Multisciences, Hangzhou, China) and PE‐conjugated anti‐mouse IgG secondary antibodies (GAM5496; Multisciences). Finally, the expression of GAS2 and CXCR4 or the expression of Calpain2 and CXCR4 were analyzed by a confocal microscope (FV1000MPE‐share; Olympus, Tokyo, Japan).

### 2.10 RNA‐seq analysis

Three biological replicates of GAS2 silenced (shGAS2) and the control (Scrambled) Jurkat cells were harvested for RNA‐seq analysis (Basepair, Suzhou, China). Differentially expressed transcripts were determined based on *P* values (Student's *t*‐test, *P* < 0.05) and fold changes (> 2). All the differentially expressed transcripts were clustered using Hierarchical Clustering.

### 2.11 Colony‐forming cell (CFC) assay

Human hematopoietic cells were plated in methylcellulose medium (MethoCult H4230, STEMCELL Technologies) supplemented with a cocktail of cytokines [SCF (50 ng·mL^−1^), IL‐3 (20 ng·mL^−1^), IL‐6 (20 ng·mL^−1^), GM‐CSF (20 ng·mL^−1^), G‐CSF (20 ng·mL^−1^), and EPO (3 IU·mL^−1^)]. Similarly, mouse bone marrow cells were isolated with Histopaque (Sigma) and then plated in methylcellulose medium (MethoCult M3231, STEMCELL Technologies) supplemented with a cocktail of cytokines [mSCF (100 ng·mL^−1^), mIL‐3 (6 ng·mL^−1^), and IL‐6 (10 ng·mL^−1^)]. Colonies were enumerated 12–14 days later.

### 2.12 Statistical analysis

All values were represented as the mean ± SEM from more than three biological replicates, and statistical analysis was performed with Student's *t*‐test, in which a *P* value < 0.05 was considered significant. Kaplan–Meier method was used to study the survival tendency, and the *P* value was estimated using the log‐rank test.

## 3 Results

### 3.1 Aberrantly expressed GAS2 promotes the growth of T‐ALL cells

**Fig. 1:**
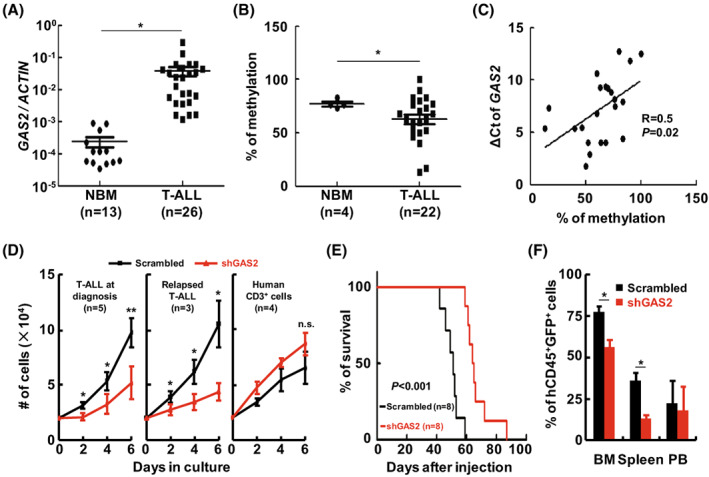
Deregulated GAS2 promotes the *in vitro* and *in vivo* growth of T‐ALL cells. (A) The bone marrow cells from T‐ALL patients (*n* = 26) and the CD3^+^ cells from Normal bone marrow (NBM) of healthy donors (*n* = 13) were collected. The expression of *GAS2* was then measured by RT‐qPCR in each sample and normalized to that of *β‐Actin*. (B) The bisulfite modified DNA samples from bone marrow cells of T‐ALL patients and normal CD3^+^ cells from healthy donors were amplified with methylation‐specific PCR to analyze the promoter region of *GAS2*, and the PCR products were subjected to sequencing. The methylation contents of bone marrow cells from T‐ALL patients (*n* = 22) and normal CD3^+^ cells (*n* = 4) were compared. (C) The correlation between DNA methylation content and the expression of *GAS2* [represented as Δ*C*_t_ (*C*_t_ (GAS2)‐*C*_t_ (β‐Actin)] was estimated. (D) Bone marrow cells from T‐ALL patients at diagnosis (*n* = 5, left panel) and those in relapse (*n* = 3, right panel) were activated with CD3/CD28 and cultured with a T cell expansion medium supplemented with IL‐2 for 2 days. Normal CD3^+^ cells from healthy donors (*n* = 4) were treated similarly as a control. These cells were transduced with lentiviral vectors for the delivery of the control (Scrambled) and shRNA against GAS2 (shGAS2). Three days later, GFP^+^ cells were isolated by FACS, and their growth analyzed. (E) GAS2 silenced cells and Scrambled Jurkat cells (8 × 10^6^ cells per mouse, eight mice in each group) were injected through tail vein into immunodeficient mice. These mice were observed closely and the survival of each group was analyzed by the Kaplan–Meier method (log‐rank test, *P* < 0.001). (F) The diseased mice from both the Scrambled and shGAS2 groups were dissected, and the leukemic cells (hCD45^+^GFP^+^) from the bone marrow (BM), spleen and peripheral blood (PB) were analyzed with flow cytometry and summarized statistically. Data were represented as the mean ± SEM, and the statistical significance was estimated with Student's *t*‐test (*P < 0.05; **P < 0.01).

To explore the functional role of GAS2 in human T‐ALL cells, the expression of GAS2 was analyzed using RT‐qPCR. The results showed that the expression of *GAS2* in bone marrow cells from T‐ALL patients was significantly higher than that in CD3^+^ cells from the normal bone marrow (NBM) of healthy donors (Fig. 1A). To explore the possible mechanism of deregulated *GAS2* expression in T‐ALL cells, the DNA methylation content in *GAS2* promoter region was analyzed, as a previous report showed that the aberrant expression of *GAS2* in CML cells was associated with DNA hypomethylation [33]. The data showed that the methylation content of *GAS2* promoter region in primary T‐ALL cells was significantly lower than that in normal CD3^+^ cells (Fig. 1B). Importantly, the transcript expression of *GAS2* correlated well with the DNA methylation content in *GAS2* promoter region (*P* = 0.02, *R* = 0.5), suggesting that hypomethylation in the promoter region of *GAS2* plays a role in the aberrant expression of *GAS2* in T‐ALL cells (Fig. 1C).

To investigate the effect of GAS2 silencing on primary T‐ALL cells, validated shGAS2 from a previous report was delivered into these cells [13], and the effect of GAS2 silencing on normal CD3^+^ cells was evaluated as a control. The results showed that GAS2 silencing significantly suppressed the growth of T‐ALL cells from newly diagnosed patients (*n* = 5) and relapsed patients (*n* = 3) (Fig. 1D); however, this action did not perturb the growth of normal CD3^+^ cells (*n* = 5). Moreover, GAS2 silencing did not alter the colony‐forming cell (CFC) production of normal CD34^+^ cells (Fig. S1). Previously, we showed that GAS2 silencing inhibits the growth of Jurkat cells [13]. In the present study, the effects of GAS2 silencing on cell cycle and apoptosis of Jurkat cells were analyzed. The data showed that GAS2 silencing significantly increased the fraction of G0/G1 cells and slightly promoted apoptosis (Figs S2 and S3).

To address the effect of GAS2 silencing on T‐cell leukemogenesis, GAS2 silenced and control Jurkat cells were injected into immunodeficient mice intravenously. Kaplan–Meier analysis indicated that GAS2 silencing significantly delayed leukemia generation (Fig. 1E), and western blot confirmed that GAS2 expression in leukemic cells (hCD45^+^GFP^+^) was lower from the GAS2 silenced group than that from the control group (Fig. S4B). The weight of the spleen in the GAS2 silenced group was significantly lower than that in the control group (Fig. S4C). In addition, flow cytometry analysis showed that infiltration of leukemic cells in both the bone marrow and spleen was significantly lower in the GAS2 silenced group than in the control group (Fig. 1F, Fig. S4D).

### 3.2 GAS2 regulates the expression of CXCR4

**Fig. 2:**
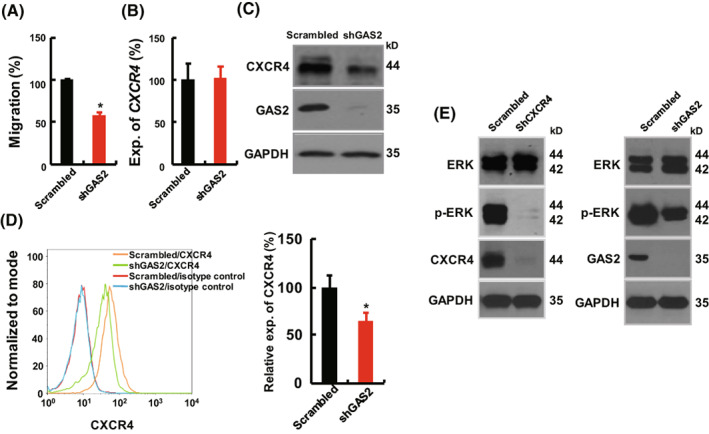
GAS2 modulates the expression of CXCR4. (A) Transwell experiment was performed to analyze the migration abilities of GAS2 silenced (shGAS2) Jurkat cells versus control (Scrambled) cells (*n* = 3). (B, C) The expression of CXCR4 in GAS2 silenced Jurkat cells versus the control cells was analyzed by RT‐qPCR (*n* = 3) and western blot, respectively. (D) The expression of CXCR4 on the cell surface was analyzed with a flow cytometer, and a representative graph was shown. The relative expression of CXCR4 (measured by mean of fluorescence, MFI) on the surface of GAS2 silenced Jurkat cells versus control cells (*n* = 3) were statistically summarized. (E) Western blots were conducted to analyze the expression of ERK and p‐ERK in Jurkat cells upon CXCR4 or GAS2 silencing. The representative results of three replicates were shown. Data were represented as the mean ± SEM, and the statistical significance was estimated with Student's *t*‐test (*P < 0.05).

To delineate the functional mechanism of GAS2 in T‐cell leukemogenesis beyond proliferation, we observed that GAS2 positively regulated the infiltration of leukemic cells *in vivo*, which allowed us to speculate that GAS2 regulates the migration of T‐ALL cells. Therefore, transwell experiments were performed and the results indicated that GAS2 silencing significantly inhibited the migration of Jurkat cells (Fig. 2A). Since the CXCL12/CXCR4 axis plays a crucial role in cell migration [34, 35], the expression of CXCR4 was analyzed. RT‐qPCR data showed that the transcript expression of *CXCR4* was not significantly changed upon GAS2 silencing (Fig. 2B), whereas western blot indicated that GAS2 silencing decreased the protein expression of CXCR4 (Fig. 2C). Moreover, cell surface expression of CXCR4 was significantly inhibited by GAS2 silencing, as measured by flow cytometry (Fig. 2D). Since ERK phosphorylation is a critical event in CXCR4 signaling [34, 35, 36], the expression of ERK and p‐ERK was analyzed. The data showed that CXCR4 silencing severely decreased the phosphorylation of ERK, whereas GAS2 silencing inhibited ERK phosphorylation to a lesser extent (Fig. 2E, Fig. S5).

**Fig. 3:**
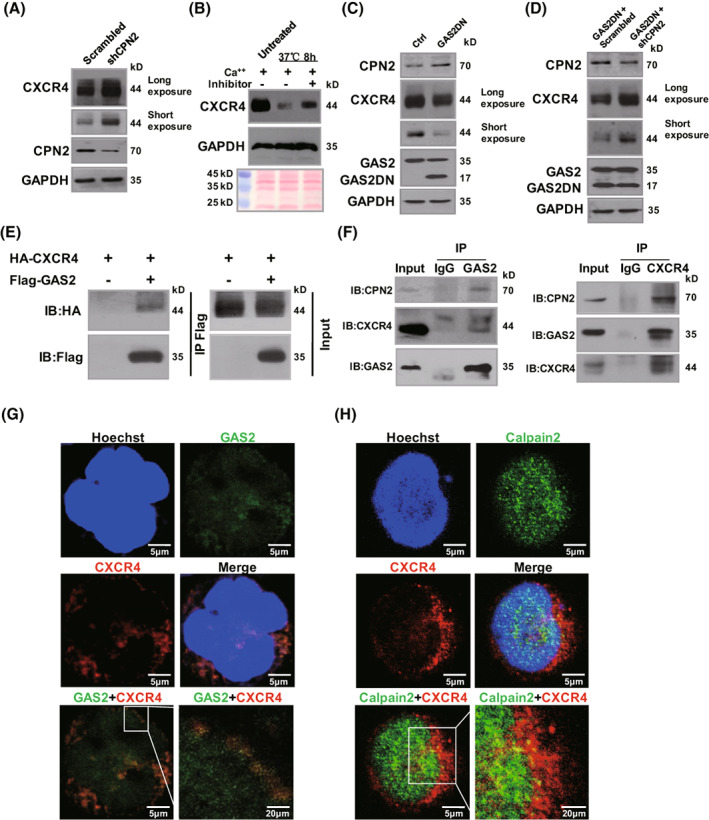
GAS2 interacts with CXCR4. (A) The expression of CXCR4 in Calpain2 silenced (shCPN2) and control (Scrambled) Jurkat cells was analyzed by western blot (*n* = 3). (B) The protein extraction of Jurkat cells was prepared. An aliquot of the extract was used as an untreated control. One aliquot of the extract was supplied with Ca^++^ only, and the other aliquot was added with both Ca^++^ and Calpain inhibitor (Z‐LLY‐FMK); both aliquots were incubated at 37 °C for 8 h. Finally, the three aliquots of lysates were separated with SDS/PAGE and transferred onto a PVDG membrane. Ponceau S staining was used to monitor the loading of the samples. The expression of CXCR4 and GAPDH was analyzed by western blot. (*n* = 4) (C) The dominant negative form of GAS2 (GAS2DN) was overexpressed in Jurkat cells, the expression of CXCR4 in these cells together with their control was analyzed by western blot (*n* = 3). (D) Calpain2 was silenced in GAS2DN overexpressed Jurkat cells, and then CXCR4 expression in these cells together with the control cells was analyzed by western blot (*n* = 3). (E) Flag‐tagged GAS2 and/or HA tagged CXCR4 were overexpressed in 293 T cells, and coimmunoprecipitation (Co‐IP) experiment was performed to analyze the interaction between GAS2 and CXCR4 (*n* = 2). (F) Immunoprecipitation (IP) against GAS2 in Jurkat cells was performed (left panel), and the expression of CXCR4 and Calpain2 (CPN2) was detected with immunoblot (IB). Conversely, IP against CXCR4 in Jurkat cells was conducted (right panel), and the expression of GAS2 and CPN2 was detected with IB (*n* = 2). (G) The expression of both GAS2 (green) and CXCR4 (red) was analyzed with confocal microscopy (*n* = 2). (H) Additionally, the expression of both Calpain2 (green) and CXCR4 (red) was analyzed with confocal microscopy (*n* = 2). The scale bar equals 5 or 20 μm as indicated in each graph.

We then explored the possible role of the GAS2/Calpain2 axis in the regulation of CXCR4 expression. The first question we addressed was whether Calpain2 regulated the endogenous expression of CXCR4 in Jurkat cells. The results showed that Calpain2 silencing (shCPN2) increased CXCR4 expression in Jurkat cells (Fig. 3A). Two aliquots of protein extraction of Jurkat cells plus Ca^++^ supplements were incubated at 37 °C for 8 h with and without a Calpain inhibitor (Z‐LLY‐FMK). Ponceau S staining was performed to confirm equal loading of these samples. Western blot showed that the expression of CXCR4, but not that of GAPDH was altered upon Ca^++^ addition (Fig. 3B). These results strongly indicated that Calpain was able to degrade CXCR4. A previous study showed that GAS2DN and GAS2 silencing had similar inhibitory effect on the growth of Jurkat cells, and the inhibitory effect of GAS2DN was partially rescued by Calpain2 silencing [13]. Herein, it was found that GAS2DN decreased CXCR4 expression as GAS2 silencing did (Fig. 3C), and Calpain2 silencing reversed the decreased expression of CXCR4 by GAS2DN (Fig. 3D). Importantly, co‐immunoprecipitation analysis showed that GAS2 and CXCR4 interacted when overexpressed in 293 T cells (Fig. 3E). Next, immunoprecipitation analysis revealed a specific interaction between endogenous CXCR4 and GAS2 in Jurkat cells (Fig. 3F, Fig. S6). Consistent with a previous report [13], Calpain2 was present in a complex containing CXCR4 and GAS2. Finally, confocal microscopy showed that GAS2 and CXCR4 were partially co‐expressed (Fig. 3G), as were Calpain2 and CXCR4 (Fig. 3H). Overall, these results indicate that endogenous GAS2 and CXCR4 interact in T‐ALL cells, providing a strong molecular basis for the regulation of CXCR4 expression by GAS2.

### 3.3 GAS2/CXCR4 axis regulates the growth of T‐ALL cells

**Fig. 4:**
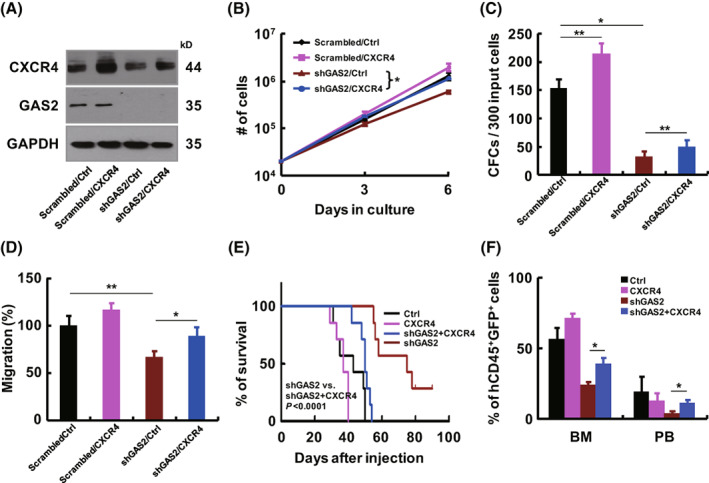
GAS2/CXCR4 axis regulates T‐cell leukemogenesis. (A–D) Jurkat cells were transduced with a combination of various lentiviruses, as indicated, and these cells were subjected to western blot, cell growth (*n* = 4), colony‐forming cell (CFC) (*n* = 4) and migration (*n* = 4) analyses. (E) Variously transduced Jurkat cells were injected into immunodeficient mice through tail vein (1 × 10^7^ cells per mouse, seven mice in each group), including control (ctrl), CXCR4, shGAS2 and shGAS2 + CXCR4 cells. The survival of each group was analyzed by the Kaplan–Meier method (log‐rank test, *P* < 0.0001). (F) The leukemic cells in the bone marrow (BM) and peripheral blood (PB) of each group of mice were analyzed by flow cytometry and summarized statistically. Data were represented as the mean ± SEM, and the statistical significance was estimated with Student's *t*‐test (*P < 0.05; **P < 0.01).

To explore the role of CXCR4 in the growth and migration regulated by GAS2, a lentiviral vector was constructed to overexpress CXCR4. Both western blot and flow cytometry confirmed that this vector elevated the expression of CXCR4 in the control (Scrambled) and GAS2 silenced (shGAS2) Jurkat cells (Fig. 4A, Fig. S7). The growth, CFC production, and migratory ability of Jurkat cells were measured to evaluate the effects of CXCR4 overexpression on Jurkat cells. The results showed that CXCR4 overexpression tended to promote the growth and migration of Jurkat cells, and significantly increased CFC production of these cells. Importantly, CXCR4 overexpression significantly enhanced the properties of GAS2 silenced Jurkat cells, particularly CFC production (Fig. 4B–D). In a xenograft model, various transduced Jurkat cells were injected into immunodeficient mice. The results showed that CXCR4 overexpression promoted T‐cell leukemogenesis, although this was not statistically significant, while CXCR4 overexpression significantly enhanced the leukemogenesis ability of GAS2 silenced Jurkat cells (Fig. 4E). Mice in the CXCR4 overexpression plus GAS2 silencing group (shGAS2 + CXCR4) had significantly more leukemic cells in the bone marrow and peripheral blood than the GAS2 silencing (shGAS2) alone group (Fig. 4F). The expression of GAS2 and CXCR4 in leukemic cells from 4 groups of mice was analyzed using western blot (Fig. S8). Overall, our data suggest that CXCR4 is partially required for GAS2 to promote T‐cell leukemogenesis, whereas overexpression of CXCR alone does not necessarily promote T‐cell leukemogenesis.

### 3.4 GAS2/CXCR4 axis regulates the expression of NOTCH1 and c‐MYC in T‐ALL cells

**Fig. 5:**
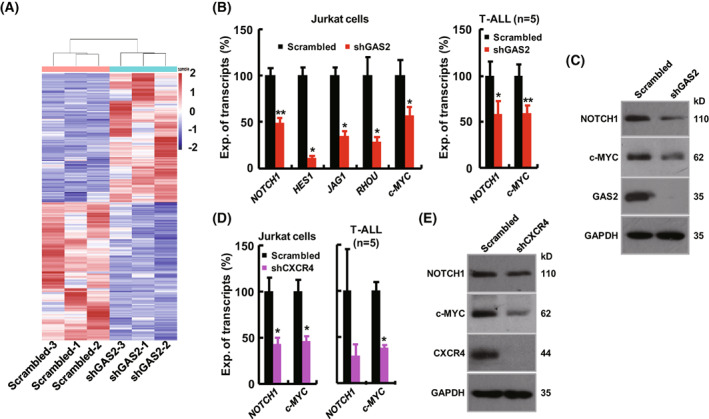
GAS2 silencing impairs NOTCH1/c‐MYC signaling in T‐ALL cells. (A) RNA‐seq data comparing GAS2 silencing (shGAS2) with control (Scrambled) Jurkat cells (*n* = 3) revealed that 249 transcripts were up‐regulated in GAS2 silenced cells compared with control cells, while 296 transcripts were down‐regulated (fold‐change ≥ 2 and *P* < 0.05), which was shown in the Heatmap. (B) RT‐qPCR was used to measure the expression of *NOTCH1*, *HES1*, *JAG1*, *RHOU* and *c‐MYC* in GAS2 silenced Jurkat cells and control cells (*n* = 4, left panel). The T‐ALL cells from patients (*n* = 5) were transduced with the control (Scrambled) and shGAS2 lentiviral vectors, and RNA samples were prepared for the gene expression analyses of both *NOTCH1* and *c‐MYC* by RT‐qPCR (right panel). (C) The protein expression of NOTCH1 and c‐MYC was analyzed in GAS2 silenced Jurkat cells versus the control cells. The representative results of four replicates were shown. (D) The expression of NOTCH1 and c‐MYC in the control and CXCR4 silenced (shCXCR4) Jurkat cells were measured by RT‐qPCR (*n* = 4, left panel), and the expression of NOTCH1 and c‐MYC in control and CXCR4 silenced (shCXCR4) primary T‐ALL cells were measured by RT‐qPCR (*n* = 5, right panel). (E) The protein expression of NOTCH1 and c‐MYC was analyzed in CXCR4 silenced Jurkat cells versus control cells. The representative results of four replicates were shown. Data were represented as the mean ± SEM, and the statistical significance was estimated with Student's *t*‐test (*P < 0.05 and **P < 0.01).

To elucidate the molecular mechanism by which the GAS2/CXCR4 axis modulates the growth of T‐ALL cells, RNA‐seq data were generated to compare GAS2 silenced Jurkat cells with the control cells (Fig. 5A, Table S6). Kyoto Encyclopedia of Genes and Genomes (KEGG) enrichment analysis suggested that Notch signaling was perturbed by GAS2 silencing (Fig. S9). Several components of Notch signaling, including NOTCH1, HES1, JAG1, and RHOU [37, 38], were chosen for validation. c‐MYC was also selected for validation, as NOTCH/c‐MYC signaling plays a crucial role in T‐ALL pathology, although the differential expression of c‐MYC was not suggested by RNA‐seq data. RT‐qPCR validated that GAS2 silencing inhibited the expression of *NOTCH1* and *c‐MYC* in both Jurkat cells and primary T‐ALL cells (Fig. 5B). Western blot showed that GAS2 silencing suppressed NOTCH and c‐MYC expression in Jurkat cells (Fig. 5C). Interestingly, CXCR4 silencing significantly suppressed the growth of primary T‐ALL cells (Fig. S10), which agreed with previous reports highlighting the critical role of CXCR4 in T‐ALL [24, 25, 26]. RT‐qPCR showed that CXCR4 silencing inhibited the expression *NOTCH1* and *c‐MYC* in both Jurkat cells and primary T‐ALL cells (Fig. 5D). Western blot showed that CXCR4 silencing decreased NOTCH and c‐MYC expression in Jurkat cells (Fig. 5E). The expression of *NOTCH1* and *c‐MYC* in bone marrow cells from T‐ALL patients compared with normal CD3^+^ cells was assessed, and the results showed that both had significantly higher expression in T‐ALL patients than in healthy donors (Fig. S11).

**Fig. 6:**
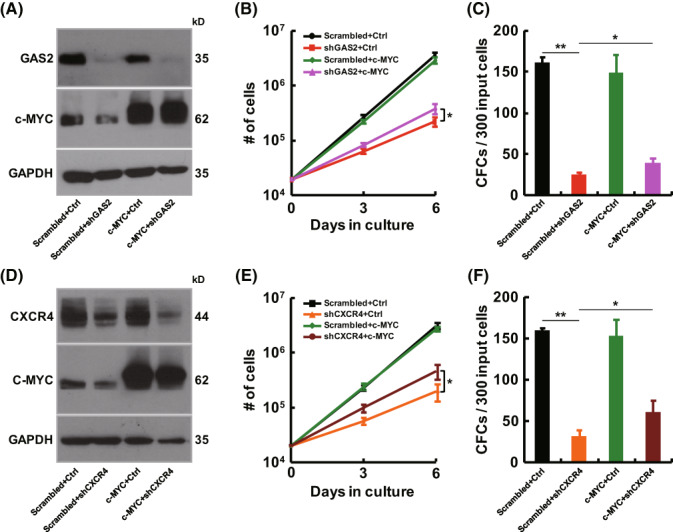
Overexpression of c‐MYC rescues the growth inhibition induced by GAS2 silencing or CXCR4 silencing. (A–C) To study the role of c‐MYC in growth inhibition induced by GAS2 silencing, Jurkat cells were transduced with a combination of various lentiviruses, as indicated. These cells were then subjected to western blot, cell growth (*n* = 4) and colony‐forming cell (CFC) (*n* = 4) assays. The representative western blot results of three replicates were shown. (D‐E) To analyze the role of c‐MYC in growth suppression induced by CXCR4 silencing, Jurkat cells were transduced with a combination of various lentiviruses, as indicated. These cells were then subjected to western blot, cell growth (*n* = 4) and colony‐forming cell (CFC) (*n* = 4) assays. The representative western blot results of two replicates were shown. Data were represented as the mean ± SEM, and the statistical significance was estimated with Student's *t*‐test (*P < 0.05 and **P < 0.01).

As the expression of c‐MYC was consistently suppressed by either GAS2 silencing or CXCR4 silencing, a rescue experiment was performed with c‐MYC overexpression. The results showed that c‐MYC overexpression partially rescued the inhibitory effects of either GAS2 silencing or CXCR4 silencing (Fig. 6), which indicated that the GAS2/CXCR4 axis partially modulated the growth of T‐ALL cells through NOTCH/c‐MYC signaling.

### 3.5 GAS2 deficiency has mild effects on normal hematopoietic cells

**Fig. 7:**
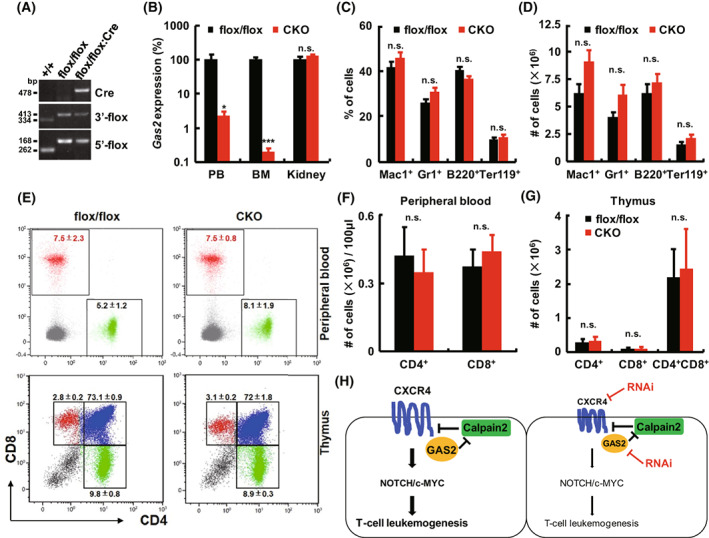
GAS2 impairment has mild effects on normal hematopoietic cells. (A) A typical photo of genotyping analyses of +/+, flox/flox and flox/flox;Vav‐iCre (conditional knockout, CKO) mice was displayed. (B) The *Gas2* transcript expression of peripheral blood (PB), bone marrow (BM), and kidney cells from both CKO (*n* = 8) and flox/flox mice (*n* = 8) was analyzed by RT‐qPCR. (C) The BM cells from 8 weeks old CKO mice (*n* = 5) and flox/flox mice (*n* = 5) were analyzed by flow cytometry for the expression of Mac‐1, Gr‐1, B220, and Ter119. (D) The absolute numbers of Mac‐1^+^, Gr‐1^+^, B220^+^, and Ter119^+^ cells from the bone marrow (2 femurs and 2 tibias) of CKO and flox/flox mice were compared. (E) The PB cells from CKO mice (*n* = 5) and flox/flox mice (*n* = 5) were analyzed for the expression of CD4 and CD8. The thymus cells from CKO mice (*n* = 5) and flox/flox mice (*n* = 5) were analyzed for the expression of CD4 and CD8 as well. (F) The absolute number of CD4^+^ and CD8^+^ cells in PB (100 μL) from CKO and flox/flox mice were compared (*n* = 5). (G) The absolute numbers of CD4^+^, CD8^+^, and CD4^+^CD8^+^ cells in the thymus from CKO and flox/flox mice were compared (*n* = 5). (H) A cartoon was illustrated to depict the role of GAS2 in T‐cell leukemogenesis. GAS2 interacts with CXCR4 and enhances its stability. This protein complex sustains the expression of NOTCH/c‐MYC and promotes the growth and invasion of T‐ALL cells. GAS2 or CXCR4 silencing suppresses the expression of NOTCH/c‐MYC to blockade T‐cell leukemogenesis. Data were represented as the mean ± SEM, and the statistical significance was estimated with Student's *t*‐test (*P < 0.05, ***P < 0.001. n.s., not significant).

To determine whether GAS2 is a suitable therapeutic target for T‐ALL, floxed and Vav‐iCre mice were crossed to study the effect of *Gas2* loss on normal murine hematopoiesis (Fig. 7A). The phenotypes of flox/flox;Vav‐iCre (conditional knockout, CKO) mice were compared with those of flox/flox mice. As expected, *Gas2* transcript expression was significantly decreased in both bone marrow and peripheral blood cells but not in kidney cells (Fig. 7B). The bone marrow samples of eight‐week old mice were analyzed by flow cytometry, and the percentage and absolute number of Mac‐1^+^, Gr‐1^+^, B220^+^, and Ter‐119^+^ cells were similar between the CKO and flox/flox groups (Fig. 7C,D). CFC assays showed that bone marrow cells from CKO and flox/flox mice had similar proliferation and differentiation capacities (Fig. S12). T‐cells from the peripheral blood and thymus were analyzed by flow cytometry, and there was no evident difference between CKO and flox/flox mice (Fig. 7E–G). The major organs of these two groups of mice were analyzed, and the coefficients (ratio of organ weight to body weight) of these organs were not significantly different (Fig. S13).

In summary, GAS2 interacts with CXCR4 and regulates the expression of CXCR4, and the GAS2/CXCR4 axis promotes T‐cell leukemogenesis. Upon GAS2 or CXCR4 silencing, T‐cell leukemogenesis was partially inhibited through NOTCH/c‐MYC signaling (Fig. 7H).

## 4 Discussion

GAS2 plays a dual role in cancer cells [5, 7, 9, 10, 11, 12, 13, 14, 15, 16]; however, most studies have not provided *in vivo* evidence. In this study, a xenograft experiment was performed, which showed that GAS2 silencing delayed leukemia generation in immunodeficient mice. In addition, GAS2 silencing significantly decreased the infiltration of leukemic cells. At the same time, we provided evidence that GAS2 silencing inhibited the growth of bone marrow cells from T‐ALL patients both at diagnosis and in relapse. Taken together, these results highlight the importance of GAS2 in T‐ALL pathogenesis.

To identify the target of GAS2/Calpain2 axis is the key to delineating the role GAS2 plays in cancer cells and how it acts. To date, only p53 and β‐catenin have been identified as targets of the GAS2/Calpain2 axis in cancer cells [7, 9]. In the present study, we found GAS2 promotes the expression of CXCR4 post‐transcriptionally. We also showed that Calpain2 decreased the endogenous expression of CXCR4. Immunoprecipitation and confocal analyses confirmed that GAS2 interacts with CXCR4. Importantly, CXCR4 overexpression alleviated the inhibitory effects of GAS2 silencing on T‐ALL cell migration and growth. Therefore, CXCR4 was identified as a novel target of the GAS2/Calpain2 axis, which deepened our understanding of this protease machinery. Nevertheless, the underlying mechanism of the direct interaction between GAS2 and CXCR4 and its biological significance remain largely unknown. It is not clear whether the interaction protects CXCR4 from degradation by limiting the access of Calpain2 to CXCR4 or whether GAS2 bound to CXCR4 simply inhibits the enzymatic activity of Calpain2.

CXCR4 overexpression has been reported in more than 20 human cancers [30, 34, 35], including T‐ALL. The development of small‐molecule inhibitors, antagonist peptides, and antibodies against CXCR4 has provided new opportunities to combat various cancers [24, 29, 36, 39, 40, 41, 42]. Therefore, understanding the regulatory mechanisms of CXCR4 in cancer cells is of great importance. Several regulatory modes of CXCR4 have been reported [25, 43, 44, 45, 46]. For instance, Calcineurin, a serine‐threonine protein phosphatase and a biomarker of T‐ALL leukemia‐initiating cells [47, 48], regulates the cell surface expression of CXCR4 in a cortactin‐dependent manner [25]. Recently, nuclear phosphofructokinase, platelet (PFKP) was reported to stimulate CXCR4 expression through c‐MYC activity in T‐ALL and lymphoma cells [26]. Therefore, the regulatory mode revealed in this study adds a new layer of complexity to CXCR4 regulation. Our study supports the notion that CXCR4 antibodies or inhibitors provide new revenue to combat T‐ALL. Our data also suggest that targeting GAS2 is a promising strategy against T‐ALL. First, the blood‐specific knockout mouse experiments demonstrated that targeting GAS2 was relatively safe. In addition, the fact that neither CXCR4 nor c‐MYC overexpression was able to fully rescue the growth inhibition induced by GAS2 silencing suggests a broad spectrum of inhibitory effects triggered by targeting GAS2 beyond CXCR4 and NOTCH/c‐MYC signaling.

Previous studies have indicated that the interplay of NOTCH and CXCR4 signaling regulates T‐ALL cells [24, 49, 50]. For example, both activated Notch1 and Notch3 promote cell surface expression of Cxcr4 in murine T‐cells [24, 49], and increased Cxcr4 expression is associated with an increased propagation of Notch3‐induced T‐ALL cells [49]. Although Cxcr4 loss in Notch1 induced T‐ALL cells led to cell death and impaired c‐Myc signaling, decreased expression of Notch1 and Myc was not observed yet [24]. In the present study, our data clearly showed that GAS2 or CXCR4 silencing inhibited the expression of both NOTCH1 and c‐MYC, and that overexpression of c‐MYC rescued the growth inhibition induced by GAS2 or CXCR4 silencing. It is worth noting that the signaling controlled by CXCR4 to regulate NOTCH/c‐MYC signaling remains elusive. For example, the question of whether ERK signaling regulated by CXCR4 participates in c‐MYC modulation is an interesting direction. Overall, our study provides new evidence of the interplay between NOTCH1/c‐MYC and CXCR4 signaling.

Our study showed that NOTCH/c‐MYC signaling was impaired upon GAS2 silencing in T‐ALL cells. However, previous reports have shown that *Gas2* loss leads to female infertility by activating Notch signaling [31, 51]. This discrepancy is likely due to cell context‐dependent effects and requires further investigations.

To date, little is known about the role of GAS2 in hematopoiesis under physiological or stressed conditions. A previous report has shown that interferon consensus sequence binding protein (Icsbp/Irf8) controls the termination of emergency granulopoiesis in response to infectious or inflammatory challenges, by repressing the transcription of both Fas‐associated phosphatase (Fap1) and Gas2 [52]. The present study showed that *Gas2* loss, specifically in the hematopoietic lineage, had a mild effect on murine hematopoiesis including T‐cell development. In addition, GAS2 silencing did not disturb the growth of normal CD3^+^ or CD34^+^ cells, which supports GAS2 as a suitable therapeutic target to eradicate T‐ALL cells or other hematological malignancies with aberrant GAS2 expression, such as CML and myeloproliferative neoplasm [12, 15, 53].

Taken together, our data show that the interaction between GAS2 and CXCR4 promotes T‐cell leukemogenesis partially through NOTCH1/c‐MYC activity, whereas GAS2 impairment does not perturb the growth of normal hematopoietic cells, which demonstrates the critical role of GAS2 in T‐ALL pathology and potentiates GAS2 as a novel therapeutic target for this disease.

## 5 Conclusions

CXCR4 has been identified as a new target of GAS2/Calpain2 in human T‐ALL cells. The GAS2/CXCR4 axis partially promotes T‐cell leukemogenesis via c‐MYC activity, whereas GAS2 impairment does not perturb normal hematopoiesis, including T‐cell development, suggesting that GAS2 is a novel therapeutic target for this disease.

## Conflict of interest

The authors declare no conflict of interest.

## Author contributions

WM, YW, JZ, JY, YW and JL performed most experimental work. HL, XH and XZ provided critical technical supports. HZ and DW supervised the quality of clinical samples. YH supervised the animal study. JW and YZ conceived the project and designed the study. WM, YW, JW and YZ wrote the manuscript. All authors read and approved the manuscript.

## Supporting information


**Fig. S1.** The effect of GAS2 silencing on the colony‐forming cell (CFC) production of normal hematopoietic CD34^+^ cells.
**Fig. S2.** The effect of GAS2 silencing on cell cycle status of Jurkat cells.
**Fig. S3.** The effect of GAS2 silencing on apoptosis of Jurkat cells.
**Fig. S4.** GAS2 silencing inhibits Jurkat cells to generate leukemia in immunodeficient mice.
**Fig. S5.** CXCR4 or GAS2 silencing inhibits ERK phosphorylation.
**Fig. S6.** Interaction between GAS2 and CXCR4 in Jurkat cells.
**Fig. S7.** The surface expression of CXCR4 in Jurkat cells upon various viral infections.
**Fig. S8.** The expression of GAS2 and CXCR4 in leukemic cells were detected.
**Fig. S9.** Kyoto Encyclopedia of Genes and Genomes (KEGG) enrichment analysis of differentially expressed transcripts comparing GAS2 silenced Jurkat cells with their control.
**Fig. S10.** CXCR4 silencing inhibits the growth of primary T‐ALL cells.
**Fig. S11.** NOTCH1 and c‐MYC are aberrantly expressed in T‐ALL patients.
**Fig. S12.** Colony‐forming cell production of the bone marrow cells from CKO and flox/flox mice.
**Fig. S13.** Gas2 depletion has no evident effects on the coefficients of major organs.
**Table S1.** The characteristics of T cell acute lymphoblastic leukemia patients recruited in this study.
**Table S2.** The primers used in this study for RT‐qPCR analysis.
**Table S3.** The antibodies used for western blot, immunofluorescence, and immunoprecipitation in this study.
**Table S4.** The shRNA sequences used in this study.
**Table S5.** The primer sequences used for the genotyping in this study.
**Table S6.** The differentially expressed transcripts upon GAS2 silencing in Jurkat cells identified by RNA‐seq.

## Data Availability

Data supporting the findings are included in this article and the Supporting information.
