# Supplementary material for: Growth arrest‐specific protein 2 (GAS2) interacts with CXCR4 to promote T‐cell leukemogenesis partially via c‐MYC
Source: Mol Oncol. 2022 Sep 11;16(20):3720–34. doi: 10.1002/1878-0261.13306 (PMC9580887; doi:10.1002/1878-0261.13306)
Supplement: Supplementary file 1 — Fig. S1. The effect of GAS2 silencing on the colony‐forming cell (CFC) production of normal hematopoietic CD34+ cells. Fig. S2. The effect of GAS2 silencing on cell cycle status of Jurkat cells. Fig. S3. The effect of GAS2 silencing on apoptosis of Jurkat cells. Fig. S4. GAS2 silencing inhibits Jurkat cells to generate leukemia in immunodeficient mice. Fig. S5. CXCR4 or GAS2 silencing inhibits ERK phosphorylation. Fig. S6. Interaction between GAS2 and CXCR4 in Jurkat cells. Fig. S7. The surface expression of CXCR4 in Jurkat cells upon various viral infections. Fig. S8. The expression of GAS2 and CXCR4 in leukemic cells were detected. Fig. S9. Kyoto Encyclopedia of Genes and Genomes (KEGG) enrichment analysis of differentially expressed transcripts comparing GAS2 silenced Jurkat cells with their control. Fig. S10. CXCR4 silencing inhibits the growth of primary T‐ALL cells. Fig. S11. NOTCH1 and c‐MYC are aberrantly expressed in T‐ALL patients. Fig. S12. Colony‐forming cell production of the bone marrow cells from CKO and flox/flox mice. Fig. S13. Gas2 depletion has no evident effects on the coefficients of major organs. Table S1. The characteristics of T cell acute lymphoblastic leukemia patients recruited in this study. Table S2. The primers used in this study for RT‐qPCR analysis. Table S3. The antibodies used for western blot, immunofluorescence, and immunoprecipitation in this study. Table S4. The shRNA sequences used in this study. Table S5. The primer sequences used for the genotyping in this study. Table S6. The differentially expressed transcripts upon GAS2 silencing in Jurkat cells identified by RNA‐seq. [file MOL2-16-3720-s001.doc]

**Supporting information**

**Growth arrest-specific 2 interacts with CXCR4 to promote T-cell leukemogenesis partially via c-MYC**

Wenjuan Ma^1,*^, Yan Wan^1,*^, Jianxiang Zhang^1^, Jianan Yao^1^, Yifei Wang^1^, Jinchang Lu^1^, Hong Liu^2,3^, Xiaorui Huang^1^, Xiuyan Zhang^1^, Haixia Zhou^2,3^, Yulong He^1,3,4,5,6^, Depei Wu^2,3,6^, Jianrong Wang^1,3,5,6,7†^, Yun Zhao^1,3,6†^

^1^ Cyrus Tang Medical Institute, Collaborative Innovation Center of Hematology, Soochow University, Suzhou 215123, China

^2^ The First Affiliated Hospital of Soochow University, Key Laboratory of Thrombosis and Hemostasis, Ministry of Health, Suzhou 215006, China

^3^ National Clinical Research Center for Hematologic Diseases, Suzhou 215006, China

^4^ Cam-Su Genomic Resources Center, Soochow University, Suzhou 215123, China

^5^ State Key Laboratory of Radiation Medicine and Radioprotection, Soochow University, Suzhou 215123, China

^6^ MOE Engineering Center of Hematological Disease, Soochow University, Suzhou 215123, China

^7^ Key Laboratory of Stem Cells and Biomedical Materials of Jiangsu Province and Chinese Ministry of Science and Technology, Suzhou 215123, China

*These authors contributed equally to this study.

^†^These authors are co-senior authors of this study.

**Supplementary Table S1.** The characteristics of T cell acute lymphoblastic leukemia patients recruited in this study.

|  | | **Patients** |
| --- | --- | --- |
| ***No.*** | ***Total*** | **31** |
|  | ***F*** | **7** |
|  | ***M*** | **24** |
| ***Age*** | ***Mean*** | **31.5** |
|  | ***Range*** | **12~62** |
|  | ***Age＜50, n(%)*** | **25(80)** |
|  | ***Age≥50, n(%)*** | **6(20)** |
| ***WBC, × 10^9^/L*** | ***Mean*** | **104.2** |
|  | ***Range*** | **2.87~369** |
|  | ***Missing, n(%)*** | **1(3.2)** |
| ***Hb, g/L*** | ***Mean*** | **90.7** |
|  | ***Range*** | **50~141** |
|  | ***Missing, n(%)*** | **1(3.2)** |
| ***Plt, × 10^9^/L*** | ***Mean*** | **66.3** |
|  | ***Range*** | **5~175** |
|  | ***Missing, n(%)*** | **1(3.2)** |
| ***Blast cells (%)*** | ***Mean*** | **76.9** |
|  | ***Range*** | **26~96** |
|  | ***Missing, n(%)*** | **1(3.2)** |

WBC, white blood cell; Hb, hemoglobin; Plt, platelet.

**Supplementary Table S2.** The primers used in this study for RT-qPCR analysis.

| **Gene Symbol** | **Sequence of primers** | **Amplicon**  **(bp)** |
| --- | --- | --- |
| *ACTB*  *(β-ACTIN)* | CACCATTGGCAATGAGCGGTTCC | 90 |
|  | GTAGTTTCGTGGATGCCACAGG |  |
| *GAS2* | GCAACCCAGAGAAGTGTGTCT | 74 |
|  | CAGGAGGCTCCACACCAT |  |
| *CXCR4* | CTCCTCTTTGTCATCACGCTTCC | 127 |
|  | GGATGAGGACACTGCTGTAGAG |  |
| *c-MYC* | CTGGTGCTCCATGAGGAGAC | 126 |
|  | AGACTCTGACCTTTTGCCAGG |  |
| *NOTCH1* | GGTGAACTGCTCTGAGGAGATC | 150 |
|  | GGATTGCAGTCGTCCACGTTGA |  |
| *HES1* | GGAAATGACAGTGAAGCACCTCC | 130 |
|  | GAAGCGGGTCACCTCGTTCATG |  |
| *JAG1* | TGCTACAACCGTGCCAGTGACT | 149 |
|  | TCAGGTGTGTCGTTGGAAGCC |  |
| *RHOU* | ACTGCCTTCGACAACTTCTCCG | 138 |
|  | GAGCAGGAAGATGTCTGTGTTGG |  |
| *Calpain2*  *(CPN2)* | GCAGCCATTGCCTCCCTCAC | 131 |
|  | ACCTCCACCCACTCGCCGTA |  |
| *β-Actin*  *(mouse)* | GAGACCTTCAACACCCCAGCCA | 92 |
|  | GAGTCCATCACAATGCCTGTG |  |
| *Gas2*  *(mouse)* | AGTTGGATAATGGTGCCTTG | 112 |
|  | GGAATCTTCTTCAGTGGTAG |  |

**Supplementary Table S3.** The antibodies used for Western blot, immunofluorescence, and immunoprecipitation in this study.

| **Antibodies** | **Information** | **Applications** |
| --- | --- | --- |
| anti-GAS2 | ab109762, Abcam, Cambridge, MA, USA | WB, IP,IF |
| anti-CXCR4 | ab124824, Abcam | WB, IP |
| anti-CXCR4 | 60042-1-Ig, Proteintech | IF |
| anti-Calpain2 | A4066, ABclonal, Wuhan, China | WB, IF |
| anti-c-MYC | ab32072, Abcam | WB |
| anti-NOTCH1 | 3608S, Cell Signaling Technology (CST), Danvers, MA, USA | WB |
| anti-ERK | 9102, CST | WB |
| anti-pERK | 4370S, CST | WB |
| anti-GAPDH | 60004-1-Ig, Proteintech, Rosemont, IL, USA | WB |
| anti-Flag | F3165, Sigma | WB, IP |
| Anti-HA | 51064-2-AP, Proteintech | WB, IP |

WB, Western blot; IF, immunofluorescence; IP, immunoprecipitation.

**Supplementary Table S4.** The shRNA sequences used in this study.

| **Genes** | **Sequences of shRNAs** |
| --- | --- |
| *GAS2* | GCAGTGAAACGAATTTCTGAA (#1) |
|  | GGTGGTCTCTGCCAGTTATAA (#2) |
| *CXCR4* | GAGAAGCATGACGGACAAGTA |
| *Calpain2* | GGAACTACCCGAACACATTCTT |

**Supplementary Table S5.** The primer sequences used for the genotyping in this study.

| **Names** | **Sequences** |
| --- | --- |
| 3’-flox | AGCTTATGGAAAACTGATGCTGTTC |
|  | TCTGAAAACTACTTTTGGAAACAAGA |
| 5’-flox | ACTGGGATCCAGTCAAACAGATCAT |
|  | CTGTTTGAGCATTGCCCTTCTGAGC |
| Cre | CTCTGACAGATGCCAGGACA |
|  | TGATTTCAGGGATGGACACA |

**Supplementary Table S6.** The differentially expressed transcripts upon GAS2 silencing in Jurkat cells identified by RNA-seq.

| **Gene ID** | **Gene name** | **Fold change** | ***P* values** |
| --- | --- | --- | --- |
| ENSG00000257073 | *AC011933.1* | 41.69 | 0.003 |
| ENSG00000234515 | *PPP1R2P1* | 36.09 | 0.034 |
| ENSG00000270069 | *RP6-99M1.2* | 35.43 | 0.000 |
| ENSG00000272477 | *RP11-158G18.1* | 31.24 | 0.037 |
| ENSG00000267526 | *RP11-178C3.6* | 30.00 | 0.047 |
| ENSG00000240720 | *LRRD1* | 24.05 | 0.011 |
| ENSG00000182873 | *RP11-181G12.2* | 18.40 | 0.022 |
| ENSG00000257242 | *C12orf79* | 18.24 | 0.036 |
| ENSG00000165182 | *CXorf58* | 17.34 | 0.003 |
| ENSG00000259780 | *RP11-304L19.12* | 15.61 | 0.001 |
| ENSG00000147606 | *SLC26A7* | 14.91 | 0.046 |
| ENSG00000211795 | *TRAV8-6* | 14.61 | 0.000 |
| ENSG00000234817 | *RP3-400B16.1* | 13.12 | 0.008 |
| ENSG00000105642 | *KCNN1* | 12.95 | 0.030 |
| ENSG00000223561 | *AC003090.1* | 12.36 | 0.012 |
| ENSG00000258448 | *RP11-109N23.5* | 12.32 | 0.037 |
| ENSG00000132259 | *CNGA4* | 11.68 | 0.021 |
| ENSG00000268471 | *MIR4453* | 10.86 | 0.003 |
| ENSG00000253744 | *AC025442.3* | 10.56 | 0.019 |
| ENSG00000272267 | *RP11-375N15.2* | 10.51 | 0.010 |
| ENSG00000237328 | *RAI1-AS1* | 10.34 | 0.011 |
| ENSG00000146376 | *ARHGAP18* | 10.28 | 0.034 |
| ENSG00000253385 | *KB-1254G8.1* | 9.54 | 0.020 |
| ENSG00000102145 | *GATA1* | 9.42 | 0.043 |
| ENSG00000261758 | *RP11-102M11.2* | 8.89 | 0.008 |
| ENSG00000135472 | *FAIM2* | 8.79 | 0.000 |
| ENSG00000170442 | *KRT86* | 8.38 | 0.008 |
| ENSG00000254266 | *RP11-594N15.2* | 7.98 | 0.011 |
| ENSG00000260896 | *RP11-314O13.1* | 7.72 | 0.006 |
| ENSG00000224843 | *LINC00240* | 7.61 | 0.040 |
| ENSG00000171951 | *SCG2* | 7.49 | 0.003 |
| ENSG00000249240 | *AC069368.3* | 7.48 | 0.043 |
| ENSG00000169715 | *MT1E* | 7.48 | 0.002 |
| ENSG00000207975 | *MIR181B1* | 7.24 | 0.019 |
| ENSG00000272223 | *RP1-20C7.6* | 7.14 | 0.002 |
| ENSG00000267219 | *AC010504.2* | 7.03 | 0.046 |
| ENSG00000174233 | *ADCY6* | 6.95 | 0.029 |
| ENSG00000118520 | *ARG1* | 6.83 | 0.049 |
| ENSG00000214773 | *RP11-717D12.1* | 6.71 | 0.025 |
| ENSG00000228133 | *AC099684.1* | 6.63 | 0.011 |
| ENSG00000228363 | *AC015971.2* | 6.58 | 0.004 |
| ENSG00000130513 | *GDF15* | 6.56 | 0.034 |
| ENSG00000173208 | *ABCD2* | 6.51 | 0.001 |
| ENSG00000213842 | *SUGT1P2* | 6.29 | 0.050 |
| ENSG00000135549 | *PKIB* | 6.22 | 0.016 |
| ENSG00000250606 | *PRSS3P2* | 6.15 | 0.000 |
| ENSG00000117707 | *PROX1* | 6.08 | 0.000 |
| ENSG00000124191 | *TOX2* | 6.04 | 0.002 |
| ENSG00000214803 | *RP11-37N22.1* | 5.99 | 0.031 |
| ENSG00000158406 | *HIST1H4H* | 5.88 | 0.009 |
| ENSG00000267681 | *CTD-3199J23.6* | 5.75 | 0.003 |
| ENSG00000273432 | *RP5-1165K10.2* | 5.37 | 0.000 |
| ENSG00000204706 | *MAMDC2-AS1* | 5.15 | 0.027 |
| ENSG00000236986 | *RP11-544A12.4* | 5.14 | 0.042 |
| ENSG00000265817 | *FSBP* | 5.01 | 0.001 |
| ENSG00000233585 | *AC115617.2* | 4.96 | 0.038 |
| ENSG00000231856 | *RP11-327P2.5* | 4.93 | 0.027 |
| ENSG00000211797 | *TRAV17* | 4.93 | 0.003 |
| ENSG00000144648 | *ACKR2* | 4.91 | 0.031 |
| ENSG00000211786 | *TRAV8-2* | 4.81 | 0.002 |
| ENSG00000236083 | *OR13E1P* | 4.75 | 0.015 |
| ENSG00000165929 | *TC2N* | 4.69 | 0.002 |
| ENSG00000172322 | *CLEC12A* | 4.51 | 0.048 |
| ENSG00000146904 | *EPHA1* | 4.45 | 0.034 |
| ENSG00000204172 | *AGAP10* | 4.43 | 0.021 |
| ENSG00000128271 | *ADORA2A* | 4.42 | 0.000 |
| ENSG00000267246 | *RP11-798G7.7* | 4.36 | 0.032 |
| ENSG00000231633 | *LINC00283* | 4.26 | 0.009 |
| ENSG00000231840 | *AC073342.12* | 4.24 | 0.040 |
| ENSG00000125245 | *GPR18* | 4.13 | 0.036 |
| ENSG00000181444 | *ZNF467* | 4.10 | 0.023 |
| ENSG00000182217 | *HIST2H4B* | 4.03 | 0.000 |
| ENSG00000160791 | *CCR5* | 4.01 | 0.001 |
| ENSG00000117318 | *ID3* | 3.99 | 0.000 |
| ENSG00000270956 | *RP11-65L3.4* | 3.94 | 0.000 |
| ENSG00000132517 | *SLC52A1* | 3.93 | 0.028 |
| ENSG00000227507 | *LTB* | 3.93 | 0.000 |
| ENSG00000230530 | *LIMD1-AS1* | 3.91 | 0.002 |
| ENSG00000121807 | *CCR2* | 3.82 | 0.006 |
| ENSG00000205930 | *C21orf49* | 3.79 | 0.021 |
| ENSG00000271721 | *RP11-337C18.9* | 3.74 | 0.022 |
| ENSG00000272211 | *RP11-347P5.1* | 3.72 | 0.028 |
| ENSG00000184678 | *HIST2H2BE* | 3.71 | 0.002 |
| ENSG00000214900 | *C14orf182* | 3.71 | 0.042 |
| ENSG00000139160 | *METTL20* | 3.63 | 0.000 |
| ENSG00000270505 | *IGHV1OR15-1* | 3.58 | 0.035 |
| ENSG00000211790 | *TRAV8-4* | 3.57 | 0.000 |
| ENSG00000176945 | *MUC20* | 3.55 | 0.000 |
| ENSG00000183941 | *HIST2H4A* | 3.53 | 0.003 |
| ENSG00000189366 | *ALG1L* | 3.51 | 0.035 |
| ENSG00000229474 | *PATL2* | 3.45 | 0.003 |
| ENSG00000232810 | *TNF* | 3.38 | 0.047 |
| ENSG00000165125 | *TRPV6* | 3.38 | 0.004 |
| ENSG00000230461 | *PROX1-AS1* | 3.38 | 0.000 |
| ENSG00000269921 | *RP11-646I6.5* | 3.37 | 0.030 |
| ENSG00000246214 | *RP11-260E18.1* | 3.35 | 0.015 |
| ENSG00000130518 | *KIAA1683* | 3.33 | 0.004 |
| ENSG00000267003 | *CTC-507E2.1* | 3.32 | 0.023 |
| ENSG00000169442 | *CD52* | 3.31 | 0.000 |
| ENSG00000143365 | *RORC* | 3.29 | 0.033 |
| ENSG00000227028 | *SLC8A1-AS1* | 3.29 | 0.044 |
| ENSG00000229164 | *TRAC* | 3.24 | 0.000 |
| ENSG00000232224 | *LINC00202-1* | 3.22 | 0.012 |
| ENSG00000211796 | *TRAV16* | 3.20 | 0.022 |
| ENSG00000113916 | *BCL6* | 3.16 | 0.000 |
| ENSG00000213613 | *RP11-380G5.3* | 3.15 | 0.001 |
| ENSG00000203739 | *RP11-296O14.3* | 3.11 | 0.013 |
| ENSG00000163823 | *CCR1* | 3.10 | 0.006 |
| ENSG00000234494 | *AC003665.1* | 3.05 | 0.007 |
| ENSG00000224897 | *POT1-AS1* | 3.04 | 0.041 |
| ENSG00000249437 | *NAIP* | 3.02 | 0.023 |
| ENSG00000092529 | *CAPN3* | 2.99 | 0.049 |
| ENSG00000007944 | *MYLIP* | 2.98 | 0.007 |
| ENSG00000272831 | *RP11-792A8.4* | 2.96 | 0.019 |
| ENSG00000171243 | *SOSTDC1* | 2.93 | 0.000 |
| ENSG00000231566 | *RP5-1158E12.3* | 2.93 | 0.036 |
| ENSG00000257524 | *RP11-203J24.9* | 2.90 | 0.009 |
| ENSG00000237672 | *KRR1P1* | 2.89 | 0.022 |
| ENSG00000271141 | *RP11-171I2.4* | 2.89 | 0.009 |
| ENSG00000009724 | *MASP2* | 2.88 | 0.000 |
| ENSG00000161082 | *CELF5* | 2.84 | 0.000 |
| ENSG00000271895 | *RP4-635E18.8* | 2.84 | 0.001 |
| ENSG00000239704 | *CDRT4* | 2.83 | 0.036 |
| ENSG00000246225 | *RP11-17A1.3* | 2.81 | 0.017 |
| ENSG00000229989 | *MIR181A1HG* | 2.77 | 0.003 |
| ENSG00000198963 | *RORB* | 2.77 | 0.002 |
| ENSG00000006534 | *ALDH3B1* | 2.77 | 0.001 |
| ENSG00000167261 | *DPEP2* | 2.77 | 0.027 |
| ENSG00000244457 | *ENO1P1* | 2.75 | 0.028 |
| ENSG00000180354 | *MTURN* | 2.74 | 0.000 |
| ENSG00000172260 | *NEGR1* | 2.72 | 0.000 |
| ENSG00000211935 | *IGHV1-3* | 2.71 | 0.001 |
| ENSG00000268947 | *AD000684.2* | 2.71 | 0.029 |
| ENSG00000176371 | *ZSCAN2* | 2.71 | 0.041 |
| ENSG00000211752 | *TRBV27* | 2.69 | 0.049 |
| ENSG00000266904 | *LINC00663* | 2.67 | 0.041 |
| ENSG00000057657 | *PRDM1* | 2.67 | 0.000 |
| ENSG00000172215 | *CXCR6* | 2.67 | 0.011 |
| ENSG00000248503 | *RP5-1000K24.2* | 2.67 | 0.026 |
| ENSG00000262663 | *RP11-497H17.1* | 2.63 | 0.029 |
| ENSG00000109927 | *TECTA* | 2.63 | 0.049 |
| ENSG00000236991 | *EDRF1-AS1* | 2.62 | 0.018 |
| ENSG00000163508 | *EOMES* | 2.61 | 0.017 |
| ENSG00000198417 | *MT1F* | 2.57 | 0.003 |
| ENSG00000261423 | *RP11-1007O24.3* | 2.57 | 0.018 |
| ENSG00000185495 | *RP11-504P24.4* | 2.56 | 0.028 |
| ENSG00000091137 | *SLC26A4* | 2.56 | 0.014 |
| ENSG00000120697 | *ALG5* | 2.55 | 0.000 |
| ENSG00000197540 | *GZMM* | 2.53 | 0.000 |
| ENSG00000113088 | *GZMK* | 2.53 | 0.003 |
| ENSG00000272588 | *RP11-440L14.4* | 2.50 | 0.024 |
| ENSG00000204653 | *ASPDH* | 2.50 | 0.000 |
| ENSG00000096996 | *IL12RB1* | 2.49 | 0.005 |
| ENSG00000124215 | *CDH26* | 2.49 | 0.044 |
| ENSG00000247950 | *SEC24B-AS1* | 2.49 | 0.021 |
| ENSG00000108821 | *COL1A1* | 2.49 | 0.014 |
| ENSG00000248019 | *FAM13A-AS1* | 2.47 | 0.004 |
| ENSG00000181722 | *ZBTB20* | 2.47 | 0.044 |
| ENSG00000180573 | *HIST1H2AC* | 2.46 | 0.023 |
| ENSG00000078487 | *ZCWPW1* | 2.46 | 0.004 |
| ENSG00000215022 | *RP1-257A7.4* | 2.45 | 0.002 |
| ENSG00000156103 | *MMP16* | 2.44 | 0.007 |
| ENSG00000125122 | *LRRC29* | 2.44 | 0.007 |
| ENSG00000231351 | *AC111200.7* | 2.43 | 0.020 |
| ENSG00000102931 | *ARL2BP* | 2.43 | 0.018 |
| ENSG00000143850 | *PLEKHA6* | 2.42 | 0.021 |
| ENSG00000261052 | *SULT1A3* | 2.42 | 0.003 |
| ENSG00000175820 | *CCDC168* | 2.40 | 0.004 |
| ENSG00000260613 | *RP3-522J7.6* | 2.40 | 0.014 |
| ENSG00000159388 | *BTG2* | 2.39 | 0.031 |
| ENSG00000255026 | *RP11-326C3.2* | 2.39 | 0.001 |
| ENSG00000090554 | *FLT3LG* | 2.39 | 0.000 |
| ENSG00000228889 | *UBAC2-AS1* | 2.39 | 0.001 |
| ENSG00000143507 | *DUSP10* | 2.37 | 0.001 |
| ENSG00000123901 | *GPR83* | 2.37 | 0.013 |
| ENSG00000121101 | *TEX14* | 2.36 | 0.021 |
| ENSG00000166289 | *PLEKHF1* | 2.36 | 0.000 |
| ENSG00000113369 | *ARRDC3* | 2.36 | 0.026 |
| ENSG00000136826 | *KLF4* | 2.35 | 0.005 |
| ENSG00000161149 | *TUBA3FP* | 2.35 | 0.036 |
| ENSG00000124762 | *CDKN1A* | 2.35 | 0.000 |
| ENSG00000268584 | *RP11-464F9.20* | 2.34 | 0.026 |
| ENSG00000246575 | *AC093162.5* | 2.34 | 0.027 |
| ENSG00000170345 | *FOS* | 2.34 | 0.023 |
| ENSG00000182809 | *CRIP2* | 2.34 | 0.044 |
| ENSG00000125968 | *ID1* | 2.32 | 0.000 |
| ENSG00000055070 | *SZRD1* | 2.32 | 0.001 |
| ENSG00000183691 | *NOG* | 2.29 | 0.011 |
| ENSG00000167554 | *ZNF610* | 2.27 | 0.003 |
| ENSG00000138381 | *ASNSD1* | 2.26 | 0.000 |
| ENSG00000156510 | *HKDC1* | 2.25 | 0.003 |
| ENSG00000176049 | *JAKMIP2* | 2.25 | 0.000 |
| ENSG00000154642 | *C21orf91* | 2.25 | 0.000 |
| ENSG00000171604 | *CXXC5* | 2.25 | 0.000 |
| ENSG00000118507 | *AKAP7* | 2.24 | 0.043 |
| ENSG00000270084 | *GAS5-AS1* | 2.24 | 0.041 |
| ENSG00000108219 | *TSPAN14* | 2.24 | 0.000 |
| ENSG00000267702 | *RP11-53B2.2* | 2.23 | 0.016 |
| ENSG00000189180 | *ZNF33A* | 2.23 | 0.041 |
| ENSG00000122877 | *EGR2* | 2.23 | 0.046 |
| ENSG00000272841 | *RP3-428L16.2* | 2.22 | 0.002 |
| ENSG00000259660 | *DNM1P47* | 2.21 | 0.002 |
| ENSG00000059804 | *SLC2A3* | 2.21 | 0.049 |
| ENSG00000058668 | *ATP2B4* | 2.20 | 0.002 |
| ENSG00000185736 | *ADARB2* | 2.20 | 0.033 |
| ENSG00000225119 | *LINC00999* | 2.19 | 0.019 |
| ENSG00000118515 | *SGK1* | 2.19 | 0.029 |
| ENSG00000203865 | *ATP1A1OS* | 2.16 | 0.027 |
| ENSG00000081320 | *STK17B* | 2.16 | 0.019 |
| ENSG00000257028 | *C15ORF37* | 2.16 | 0.032 |
| ENSG00000109929 | *SC5D* | 2.14 | 0.004 |
| ENSG00000168502 | *SOGA2* | 2.14 | 0.000 |
| ENSG00000109184 | *DCUN1D4* | 2.14 | 0.004 |
| ENSG00000246308 | *RP11-685M7.3* | 2.13 | 0.036 |
| ENSG00000185338 | *SOCS1* | 2.13 | 0.004 |
| ENSG00000110848 | *CD69* | 2.13 | 0.001 |
| ENSG00000230359 | *TPI1P2* | 2.13 | 0.044 |
| ENSG00000256586 | *AC091171.1* | 2.12 | 0.037 |
| ENSG00000119661 | *DNAL1* | 2.12 | 0.046 |
| ENSG00000175879 | *HOXD8* | 2.12 | 0.012 |
| ENSG00000246090 | *RP11-696N14.1* | 2.11 | 0.007 |
| ENSG00000271646 | *RP11-326I11.3* | 2.11 | 0.001 |
| ENSG00000254510 | *RP11-867G23.10* | 2.11 | 0.001 |
| ENSG00000166341 | *DCHS1* | 2.10 | 0.035 |
| ENSG00000162825 | *NBPF8* | 2.10 | 0.001 |
| ENSG00000206341 | *HLA-H* | 2.09 | 0.021 |
| ENSG00000156299 | *TIAM1* | 2.09 | 0.000 |
| ENSG00000161040 | *FBXL13* | 2.08 | 0.038 |
| ENSG00000168679 | *SLC16A4* | 2.08 | 0.042 |
| ENSG00000183979 | *NPB* | 2.07 | 0.000 |
| ENSG00000135482 | *ZC3H10* | 2.07 | 0.005 |
| ENSG00000261324 | *RP11-174G6.5* | 2.07 | 0.003 |
| ENSG00000260805 | *RP11-61J19.4* | 2.07 | 0.014 |
| ENSG00000234773 | *CTD-2666L21.1* | 2.06 | 0.046 |
| ENSG00000237094 | *RP4-669L17.10* | 2.06 | 0.035 |
| ENSG00000139714 | *MORN3* | 2.06 | 0.026 |
| ENSG00000214960 | *ISPD* | 2.06 | 0.044 |
| ENSG00000268852 | *AC132872.2* | 2.06 | 0.034 |
| ENSG00000024422 | *EHD2* | 2.06 | 0.024 |
| ENSG00000271816 | *RP11-574K11.28* | 2.05 | 0.003 |
| ENSG00000204934 | *ATP6V0E2-AS1* | 2.04 | 0.001 |
| ENSG00000178233 | *TMEM151B* | 2.04 | 0.000 |
| ENSG00000163701 | *IL17RE* | 2.03 | 0.002 |
| ENSG00000258881 | *AC007040.11* | 2.03 | 0.000 |
| ENSG00000266972 | *LRRC37A9P* | 2.03 | 0.001 |
| ENSG00000185507 | *IRF7* | 2.02 | 0.000 |
| ENSG00000223797 | *ENTPD3-AS1* | 2.02 | 0.024 |
| ENSG00000249825 | *CTD-2201I18.1* | 2.01 | 0.011 |
| ENSG00000132819 | *RBM38* | 2.00 | 0.035 |
| ENSG00000113600 | *C9* | 0.50 | 0.000 |
| ENSG00000011347 | *SYT7* | 0.50 | 0.012 |
| ENSG00000148400 | *NOTCH1* | 0.50 | 0.000 |
| ENSG00000198435 | *NRARP* | 0.50 | 0.006 |
| ENSG00000125462 | *C1orf61* | 0.50 | 0.016 |
| ENSG00000205047 | *FLJ00104* | 0.50 | 0.000 |
| ENSG00000003137 | *CYP26B1* | 0.50 | 0.004 |
| ENSG00000129159 | *KCNC1* | 0.50 | 0.013 |
| ENSG00000257086 | *RP11-783K16.13* | 0.50 | 0.012 |
| ENSG00000215298 | *FP15737* | 0.50 | 0.022 |
| ENSG00000167880 | *EVPL* | 0.49 | 0.000 |
| ENSG00000119408 | *NEK6* | 0.49 | 0.000 |
| ENSG00000169398 | *PTK2* | 0.49 | 0.002 |
| ENSG00000273014 | *RP11-225B17.2* | 0.49 | 0.038 |
| ENSG00000154118 | *JPH3* | 0.49 | 0.010 |
| ENSG00000150768 | *DLAT* | 0.49 | 0.000 |
| ENSG00000103257 | *SLC7A5* | 0.49 | 0.005 |
| ENSG00000162543 | *UBXN10* | 0.49 | 0.000 |
| ENSG00000058262 | *SEC61A1* | 0.49 | 0.000 |
| ENSG00000171316 | *CHD7* | 0.49 | 0.000 |
| ENSG00000175497 | *DPP10* | 0.49 | 0.000 |
| ENSG00000249485 | *RBBP4P1* | 0.49 | 0.000 |
| ENSG00000135144 | *DTX1* | 0.49 | 0.000 |
| ENSG00000082153 | *BZW1* | 0.49 | 0.000 |
| ENSG00000182319 | *SGK223* | 0.49 | 0.030 |
| ENSG00000076641 | *PAG1* | 0.48 | 0.000 |
| ENSG00000229119 | *CTB-63M22.1* | 0.48 | 0.000 |
| ENSG00000118985 | *ELL2* | 0.48 | 0.004 |
| ENSG00000162341 | *TPCN2* | 0.48 | 0.000 |
| ENSG00000117425 | *PTCH2* | 0.48 | 0.041 |
| ENSG00000260359 | *RP11-4F5.2* | 0.48 | 0.006 |
| ENSG00000171119 | *NRTN* | 0.48 | 0.031 |
| ENSG00000171208 | *NETO2* | 0.48 | 0.000 |
| ENSG00000166411 | *IDH3A* | 0.48 | 0.009 |
| ENSG00000133477 | *FAM83F* | 0.48 | 0.002 |
| ENSG00000085365 | *SCAMP1* | 0.48 | 0.000 |
| ENSG00000140367 | *UBE2Q2* | 0.48 | 0.027 |
| ENSG00000147206 | *NXF3* | 0.48 | 0.041 |
| ENSG00000198561 | *CTNND1* | 0.47 | 0.000 |
| ENSG00000136895 | *GARNL3* | 0.47 | 0.005 |
| ENSG00000173868 | *PHOSPHO1* | 0.47 | 0.004 |
| ENSG00000267160 | *RP11-1072C15.4* | 0.47 | 0.010 |
| ENSG00000163293 | *NIPAL1* | 0.47 | 0.034 |
| ENSG00000145864 | *GABRB2* | 0.47 | 0.002 |
| ENSG00000068793 | *CYFIP1* | 0.47 | 0.000 |
| ENSG00000070159 | *PTPN3* | 0.47 | 0.000 |
| ENSG00000138378 | *STAT4* | 0.47 | 0.049 |
| ENSG00000198053 | *SIRPA* | 0.47 | 0.023 |
| ENSG00000175175 | *PPM1E* | 0.47 | 0.000 |
| ENSG00000135776 | *ABCB10* | 0.47 | 0.000 |
| ENSG00000105514 | *RAB3D* | 0.47 | 0.029 |
| ENSG00000184302 | *SIX6* | 0.47 | 0.000 |
| ENSG00000120833 | *SOCS2* | 0.46 | 0.024 |
| ENSG00000146094 | *DOK3* | 0.46 | 0.021 |
| ENSG00000180758 | *GPR157* | 0.46 | 0.003 |
| ENSG00000102038 | *SMARCA1* | 0.46 | 0.011 |
| ENSG00000084090 | *STARD7* | 0.46 | 0.000 |
| ENSG00000235636 | *NUS1P1* | 0.46 | 0.001 |
| ENSG00000089157 | *RPLP0* | 0.46 | 0.000 |
| ENSG00000183853 | *KIRREL* | 0.46 | 0.000 |
| ENSG00000106018 | *VIPR2* | 0.46 | 0.032 |
| ENSG00000122035 | *RASL11A* | 0.46 | 0.000 |
| ENSG00000131238 | *PPT1* | 0.46 | 0.002 |
| ENSG00000171914 | *TLN2* | 0.46 | 0.000 |
| ENSG00000168280 | *KIF5C* | 0.46 | 0.036 |
| ENSG00000108830 | *RND2* | 0.46 | 0.002 |
| ENSG00000100711 | *ZFYVE21* | 0.46 | 0.000 |
| ENSG00000136143 | *SUCLA2* | 0.46 | 0.000 |
| ENSG00000196782 | *MAML3* | 0.45 | 0.047 |
| ENSG00000076554 | *TPD52* | 0.45 | 0.000 |
| ENSG00000136213 | *CHST12* | 0.45 | 0.000 |
| ENSG00000158163 | *DZIP1L* | 0.45 | 0.045 |
| ENSG00000261534 | *RP11-244O19.1* | 0.45 | 0.001 |
| ENSG00000166482 | *MFAP4* | 0.45 | 0.002 |
| ENSG00000125533 | *BHLHE23* | 0.45 | 0.000 |
| ENSG00000166342 | *NETO1* | 0.45 | 0.000 |
| ENSG00000117877 | *CD3EAP* | 0.45 | 0.010 |
| ENSG00000092067 | *CEBPE* | 0.45 | 0.004 |
| ENSG00000104728 | *ARHGEF10* | 0.45 | 0.001 |
| ENSG00000187017 | *ESPN* | 0.45 | 0.002 |
| ENSG00000157782 | *CABP1* | 0.45 | 0.002 |
| ENSG00000117519 | *CNN3* | 0.45 | 0.000 |
| ENSG00000101384 | *JAG1* | 0.45 | 0.001 |
| ENSG00000110492 | *MDK* | 0.45 | 0.000 |
| ENSG00000137834 | *SMAD6* | 0.44 | 0.000 |
| ENSG00000080493 | *SLC4A4* | 0.44 | 0.006 |
| ENSG00000167994 | *RAB3IL1* | 0.44 | 0.000 |
| ENSG00000101935 | *AMMECR1* | 0.44 | 0.000 |
| ENSG00000043355 | *ZIC2* | 0.44 | 0.000 |
| ENSG00000187122 | *SLIT1* | 0.44 | 0.001 |
| ENSG00000203401 | *AC009061.1* | 0.44 | 0.006 |
| ENSG00000160216 | *AGPAT3* | 0.44 | 0.000 |
| ENSG00000164647 | *STEAP1* | 0.44 | 0.000 |
| ENSG00000111664 | *GNB3* | 0.44 | 0.000 |
| ENSG00000213553 | *RPLP0P6* | 0.44 | 0.000 |
| ENSG00000240906 | *AP000356.1* | 0.43 | 0.012 |
| ENSG00000170577 | *SIX2* | 0.43 | 0.020 |
| ENSG00000185989 | *RASA3* | 0.43 | 0.017 |
| ENSG00000269404 | *SPIB* | 0.43 | 0.012 |
| ENSG00000125510 | *OPRL1* | 0.43 | 0.002 |
| ENSG00000122223 | *CD244* | 0.43 | 0.000 |
| ENSG00000111110 | *PPM1H* | 0.42 | 0.000 |
| ENSG00000235033 | *RP11-61I13.3* | 0.42 | 0.029 |
| ENSG00000124942 | *AHNAK* | 0.42 | 0.006 |
| ENSG00000168952 | *STXBP6* | 0.42 | 0.002 |
| ENSG00000169884 | *WNT10B* | 0.42 | 0.005 |
| ENSG00000055118 | *KCNH2* | 0.42 | 0.000 |
| ENSG00000165629 | *ATP5C1* | 0.42 | 0.000 |
| ENSG00000133106 | *EPSTI1* | 0.42 | 0.000 |
| ENSG00000272789 | *RP11-286H15.1* | 0.42 | 0.000 |
| ENSG00000070731 | *ST6GALNAC2* | 0.42 | 0.041 |
| ENSG00000215915 | *ATAD3C* | 0.42 | 0.015 |
| ENSG00000182871 | *COL18A1* | 0.41 | 0.001 |
| ENSG00000118971 | *CCND2* | 0.41 | 0.001 |
| ENSG00000127528 | *KLF2* | 0.41 | 0.001 |
| ENSG00000241962 | *C2orf15* | 0.41 | 0.013 |
| ENSG00000148488 | *ST8SIA6* | 0.41 | 0.047 |
| ENSG00000196739 | *COL27A1* | 0.40 | 0.000 |
| ENSG00000140538 | *NTRK3* | 0.40 | 0.000 |
| ENSG00000266189 | *MIR3186* | 0.40 | 0.009 |
| ENSG00000269242 | *CTD-2192J16.22* | 0.40 | 0.001 |
| ENSG00000154734 | *ADAMTS1* | 0.40 | 0.000 |
| ENSG00000188290 | *HES4* | 0.40 | 0.000 |
| ENSG00000148344 | *PTGES* | 0.40 | 0.023 |
| ENSG00000267742 | *FAM60CP* | 0.40 | 0.009 |
| ENSG00000138772 | *ANXA3* | 0.40 | 0.005 |
| ENSG00000197965 | *MPZL1* | 0.40 | 0.000 |
| ENSG00000104722 | *NEFM* | 0.39 | 0.047 |
| ENSG00000165383 | *LRRC18* | 0.39 | 0.026 |
| ENSG00000101194 | *SLC17A9* | 0.39 | 0.029 |
| ENSG00000145431 | *PDGFC* | 0.39 | 0.000 |
| ENSG00000181418 | *DDN* | 0.39 | 0.034 |
| ENSG00000168904 | *LRRC28* | 0.39 | 0.000 |
| ENSG00000136848 | *DAB2IP* | 0.39 | 0.012 |
| ENSG00000129521 | *EGLN3* | 0.39 | 0.035 |
| ENSG00000150681 | *RGS18* | 0.39 | 0.000 |
| ENSG00000131477 | *RAMP2* | 0.38 | 0.007 |
| ENSG00000114200 | *BCHE* | 0.38 | 0.000 |
| ENSG00000167986 | *DDB1* | 0.38 | 0.000 |
| ENSG00000125089 | *SH3TC1* | 0.38 | 0.000 |
| ENSG00000108406 | *DHX40* | 0.38 | 0.000 |
| ENSG00000246985 | *SOCS2-AS1* | 0.38 | 0.021 |
| ENSG00000187554 | *TLR5* | 0.38 | 0.001 |
| ENSG00000153989 | *NUS1* | 0.38 | 0.000 |
| ENSG00000120549 | *KIAA1217* | 0.37 | 0.000 |
| ENSG00000262877 | *RP11-1055B8.4* | 0.37 | 0.035 |
| ENSG00000130707 | *ASS1* | 0.37 | 0.046 |
| ENSG00000155893 | *ACPL2* | 0.37 | 0.000 |
| ENSG00000179399 | *GPC5* | 0.37 | 0.000 |
| ENSG00000150637 | *CD226* | 0.37 | 0.023 |
| ENSG00000054793 | *ATP9A* | 0.36 | 0.008 |
| ENSG00000127585 | *FBXL16* | 0.36 | 0.000 |
| ENSG00000253368 | *TRNP1* | 0.36 | 0.000 |
| ENSG00000170989 | *S1PR1* | 0.36 | 0.000 |
| ENSG00000164520 | *RAET1E* | 0.36 | 0.007 |
| ENSG00000172005 | *MAL* | 0.36 | 0.003 |
| ENSG00000204335 | *SP5* | 0.36 | 0.013 |
| ENSG00000155307 | *SAMSN1* | 0.35 | 0.000 |
| ENSG00000168004 | *HRASLS5* | 0.35 | 0.008 |
| ENSG00000149218 | *ENDOD1* | 0.35 | 0.000 |
| ENSG00000133216 | *EPHB2* | 0.35 | 0.004 |
| ENSG00000082146 | *STRADB* | 0.35 | 0.000 |
| ENSG00000152766 | *ANKRD22* | 0.35 | 0.014 |
| ENSG00000267665 | *RP11-13K12.2* | 0.35 | 0.027 |
| ENSG00000158473 | *CD1D* | 0.35 | 0.000 |
| ENSG00000130283 | *GDF1* | 0.34 | 0.023 |
| ENSG00000243323 | *PTPRVP* | 0.34 | 0.031 |
| ENSG00000196460 | *RFX8* | 0.34 | 0.000 |
| ENSG00000270689 | *RP11-712B9.4* | 0.34 | 0.027 |
| ENSG00000107447 | *DNTT* | 0.34 | 0.011 |
| ENSG00000165449 | *SLC16A9* | 0.33 | 0.000 |
| ENSG00000186827 | *TNFRSF4* | 0.33 | 0.000 |
| ENSG00000227459 | *AC079612.2* | 0.33 | 0.035 |
| ENSG00000179934 | *CCR8* | 0.32 | 0.000 |
| ENSG00000183570 | *PCBP3* | 0.32 | 0.002 |
| ENSG00000267957 | *RP11-178G16.4* | 0.32 | 0.001 |
| ENSG00000272512 | *RP11-54O7.17* | 0.31 | 0.001 |
| ENSG00000189410 | *SH2D5* | 0.31 | 0.000 |
| ENSG00000242575 | *AC012501.3* | 0.31 | 0.000 |
| ENSG00000107282 | *APBA1* | 0.31 | 0.020 |
| ENSG00000225986 | *UBXN10-AS1* | 0.31 | 0.001 |
| ENSG00000089472 | *HEPH* | 0.30 | 0.023 |
| ENSG00000141449 | *GREB1L* | 0.30 | 0.039 |
| ENSG00000116574 | *RHOU* | 0.30 | 0.000 |
| ENSG00000163673 | *DCLK3* | 0.30 | 0.001 |
| ENSG00000260007 | *RP11-315D16.2* | 0.29 | 0.010 |
| ENSG00000176320 | *RP11-404O13.5* | 0.29 | 0.000 |
| ENSG00000110427 | *KIAA1549L* | 0.29 | 0.000 |
| ENSG00000106809 | *OGN* | 0.29 | 0.001 |
| ENSG00000115380 | *EFEMP1* | 0.29 | 0.000 |
| ENSG00000158485 | *CD1B* | 0.29 | 0.000 |
| ENSG00000267104 | *TBC1D3P1-DHX40P1* | 0.29 | 0.019 |
| ENSG00000132437 | *DDC* | 0.29 | 0.015 |
| ENSG00000235105 | *RP11-329A14.1* | 0.28 | 0.002 |
| ENSG00000229914 | *RP11-404O13.4* | 0.28 | 0.000 |
| ENSG00000228168 | *HNRNPA1P21* | 0.28 | 0.000 |
| ENSG00000116690 | *PRG4* | 0.28 | 0.011 |
| ENSG00000128322 | *IGLL1* | 0.28 | 0.000 |
| ENSG00000196758 | *AC079612.1* | 0.28 | 0.001 |
| ENSG00000101311 | *FERMT1* | 0.28 | 0.020 |
| ENSG00000168405 | *CMAHP* | 0.27 | 0.000 |
| ENSG00000197430 | *OPALIN* | 0.27 | 0.003 |
| ENSG00000262921 | *RP11-141J13.3* | 0.27 | 0.009 |
| ENSG00000141433 | *ADCYAP1* | 0.27 | 0.000 |
| ENSG00000107831 | *FGF8* | 0.26 | 0.016 |
| ENSG00000183508 | *FAM46C* | 0.26 | 0.000 |
| ENSG00000196419 | *XRCC6* | 0.26 | 0.000 |
| ENSG00000158477 | *CD1A* | 0.26 | 0.000 |
| ENSG00000260807 | *RP11-161M6.2* | 0.26 | 0.000 |
| ENSG00000100450 | *GZMH* | 0.26 | 0.002 |
| ENSG00000242252 | *BGLAP* | 0.26 | 0.013 |
| ENSG00000149451 | *ADAM33* | 0.26 | 0.028 |
| ENSG00000169758 | *C15orf27* | 0.26 | 0.025 |
| ENSG00000167807 | *CTD-2369P2.10* | 0.25 | 0.011 |
| ENSG00000128918 | *ALDH1A2* | 0.25 | 0.000 |
| ENSG00000109906 | *ZBTB16* | 0.25 | 0.043 |
| ENSG00000151892 | *GFRA1* | 0.25 | 0.001 |
| ENSG00000119401 | *TRIM32* | 0.25 | 0.000 |
| ENSG00000100448 | *CTSG* | 0.25 | 0.000 |
| ENSG00000001630 | *CYP51A1* | 0.24 | 0.000 |
| ENSG00000176406 | *RIMS2* | 0.24 | 0.000 |
| ENSG00000227400 | *AC012501.2* | 0.24 | 0.001 |
| ENSG00000180878 | *C11orf42* | 0.24 | 0.019 |
| ENSG00000128342 | *LIF* | 0.23 | 0.016 |
| ENSG00000223631 | *LINC01120* | 0.23 | 0.023 |
| ENSG00000177311 | *ZBTB38* | 0.22 | 0.026 |
| ENSG00000152268 | *SPON1* | 0.22 | 0.013 |
| ENSG00000076716 | *GPC4* | 0.22 | 0.000 |
| ENSG00000129682 | *FGF13* | 0.22 | 0.001 |
| ENSG00000256377 | *RP11-1060J15.4* | 0.21 | 0.046 |
| ENSG00000249550 | *RP11-438N16.1* | 0.21 | 0.044 |
| ENSG00000257527 | *MIR3179-3* | 0.20 | 0.004 |
| ENSG00000132746 | *ALDH3B2* | 0.20 | 0.013 |
| ENSG00000158488 | *CD1E* | 0.20 | 0.000 |
| ENSG00000076248 | *UNG* | 0.20 | 0.000 |
| ENSG00000215113 | *CXorf49B* | 0.19 | 0.018 |
| ENSG00000176659 | *C20orf197* | 0.19 | 0.000 |
| ENSG00000137177 | *KIF13A* | 0.19 | 0.011 |
| ENSG00000268655 | *CTB-60B18.10* | 0.19 | 0.020 |
| ENSG00000158481 | *CD1C* | 0.19 | 0.000 |
| ENSG00000244617 | *ASPRV1* | 0.19 | 0.017 |
| ENSG00000261705 | *RP11-61A14.2* | 0.19 | 0.000 |
| ENSG00000233355 | *CHRM3-AS2* | 0.18 | 0.049 |
| ENSG00000254979 | *RP11-872D17.8* | 0.18 | 0.006 |
| ENSG00000114315 | *HES1* | 0.18 | 0.001 |
| ENSG00000184596 | *AF207550.1* | 0.17 | 0.005 |
| ENSG00000163157 | *TMOD4* | 0.17 | 0.022 |
| ENSG00000225873 | *LINC00694* | 0.17 | 0.000 |
| ENSG00000064201 | *TSPAN32* | 0.17 | 0.036 |
| ENSG00000266992 | *DHX40P1* | 0.17 | 0.000 |
| ENSG00000115590 | *IL1R2* | 0.16 | 0.019 |
| ENSG00000178460 | *MCMDC2* | 0.16 | 0.021 |
| ENSG00000197632 | *SERPINB2* | 0.16 | 0.000 |
| ENSG00000065609 | *SNAP91* | 0.16 | 0.005 |
| ENSG00000168671 | *UGT3A2* | 0.15 | 0.043 |
| ENSG00000134532 | *SOX5* | 0.15 | 0.015 |
| ENSG00000255949 | *AP003419.16* | 0.15 | 0.000 |
| ENSG00000146205 | *ANO7* | 0.15 | 0.010 |
| ENSG00000156414 | *TDRD9* | 0.15 | 0.000 |
| ENSG00000147647 | *DPYS* | 0.15 | 0.018 |
| ENSG00000138162 | *TACC2* | 0.14 | 0.033 |
| ENSG00000236656 | *RP11-144L1.4* | 0.14 | 0.001 |
| ENSG00000255594 | *Z82188.1* | 0.14 | 0.014 |
| ENSG00000206052 | *DOK6* | 0.13 | 0.011 |
| ENSG00000215115 | *CXorf49* | 0.13 | 0.000 |
| ENSG00000248394 | *FOSL1P1* | 0.13 | 0.037 |
| ENSG00000004809 | *SLC22A16* | 0.13 | 0.002 |
| ENSG00000138356 | *AOX1* | 0.13 | 0.021 |
| ENSG00000266088 | *RP5-1028K7.2* | 0.12 | 0.004 |
| ENSG00000264187 | *RP11-45M22.4* | 0.12 | 0.033 |
| ENSG00000206181 | *TCEB3B* | 0.11 | 0.047 |
| ENSG00000233922 | *AL133493.2* | 0.11 | 0.002 |
| ENSG00000157214 | *STEAP2* | 0.11 | 0.038 |
| ENSG00000226644 | *RP11-128M1.1* | 0.10 | 0.028 |
| ENSG00000196126 | *HLA-DRB1* | 0.09 | 0.000 |
| ENSG00000165029 | *ABCA1* | 0.09 | 0.000 |
| ENSG00000273155 | *MRPL30* | 0.09 | 0.009 |
| ENSG00000148935 | *GAS2* | 0.08 | 0.000 |
| ENSG00000117322 | *CR2* | 0.08 | 0.000 |
| ENSG00000183032 | *SLC25A21* | 0.08 | 0.039 |
| ENSG00000038295 | *TLL1* | 0.08 | 0.005 |
| ENSG00000272934 | *RP11-392E22.10* | 0.08 | 0.006 |
| ENSG00000249428 | *RP11-503N18.3* | 0.07 | 0.024 |
| ENSG00000168481 | *LGI3* | 0.07 | 0.001 |
| ENSG00000203305 | *AC010525.1* | 0.07 | 0.014 |
| ENSG00000100453 | *GZMB* | 0.06 | 0.012 |
| ENSG00000233639 | *LINC01158* | 0.06 | 0.006 |
| ENSG00000095585 | *BLNK* | 0.05 | 0.018 |
| ENSG00000257034 | *AL021707.2* | 0.05 | 0.011 |
| ENSG00000132854 | *KANK4* | 0.05 | 0.004 |
| ENSG00000229530 | *RP11-62C3.8* | 0.04 | 0.023 |
| ENSG00000237382 | *RPL21P121* | 0.03 | 0.017 |
| ENSG00000233163 | *RP11-259F16.3* | 0.03 | 0.039 |
| ENSG00000163554 | *SPTA1* | 0.03 | 0.004 |
| ENSG00000216906 | *RP11-350J20.9* | 0.02 | 0.020 |
| ENSG00000240770 | *C21orf91-OT1* | 0.02 | 0.000 |


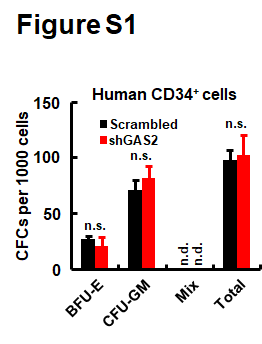


**Supplementary Fig. S1.** The effect of GAS2 silencing on the colony-forming cell (CFC) production of normal hematopoietic CD34^+^ cells. The CD34^+^ cells were transduced with the control (Scrambled) and shGAS2 lentiviral vectors. The transduced cells were then subjected to CFC assay (n=3). BFU-E, burst-forming unit-erythroid; CFU-GM, colony-forming unit-granulocyte/macrophage; Mix, colony-forming unit-granulocyte, erythroid, macrophage, megakaryocyte. Data were represented as the mean ± SEM, and the statistical significance was estimated with Student’s *t*-test. n.d., not detected.


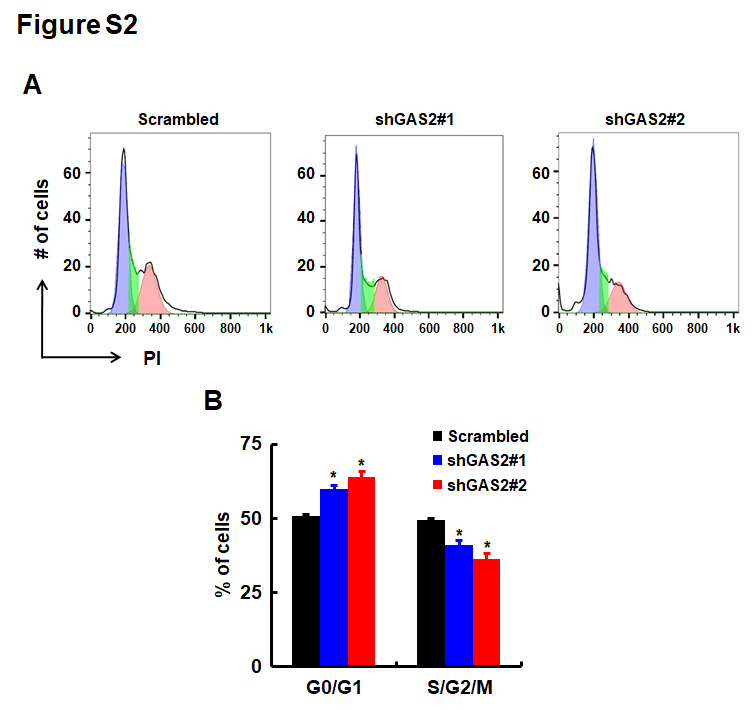


**Supplementary Fig. S2.** The effect of GAS2 silencing on cell cycle status of Jurkat cells. The control (Scrambled) and GAS2 silenced (shGAS2#1 and shGAS2#2) Jurkat cells were analyzed for their cell cycle status (n=3). (A) The representative photos were shown. (B) The results were analyzed statistically. Data were represented as the mean ± SEM, and the statistical significance was estimated with Student’s *t*-test. (**P*<0.05)


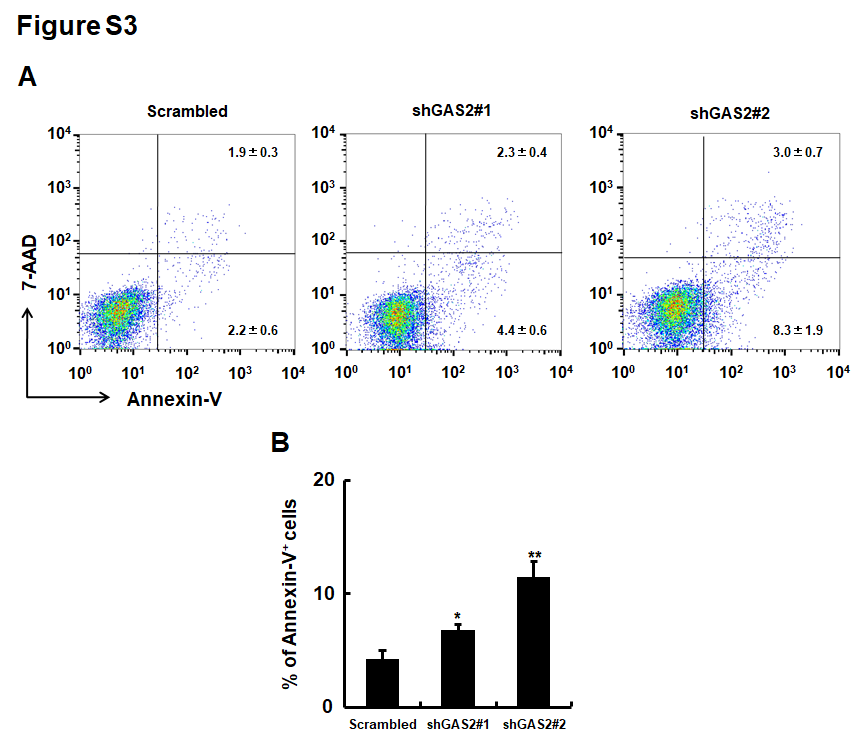


**Supplementary Fig. S3.** The effect of GAS2 silencing on apoptosis of Jurkat cells. The apoptosis of the control (Scrambled) and GAS2 silenced (shGAS2#1 and shGAS2#2) Jurkat cells were analyzed by Annexin-V/7-AAD staining (n=4). (A) The representative graphs were shown. (B) The results were analyzed statistically. Data were represented as the mean ± SEM, and the statistical significance was estimated with Student’s *t*-test. (**P*<0.05 and ***P*<0.01)

**
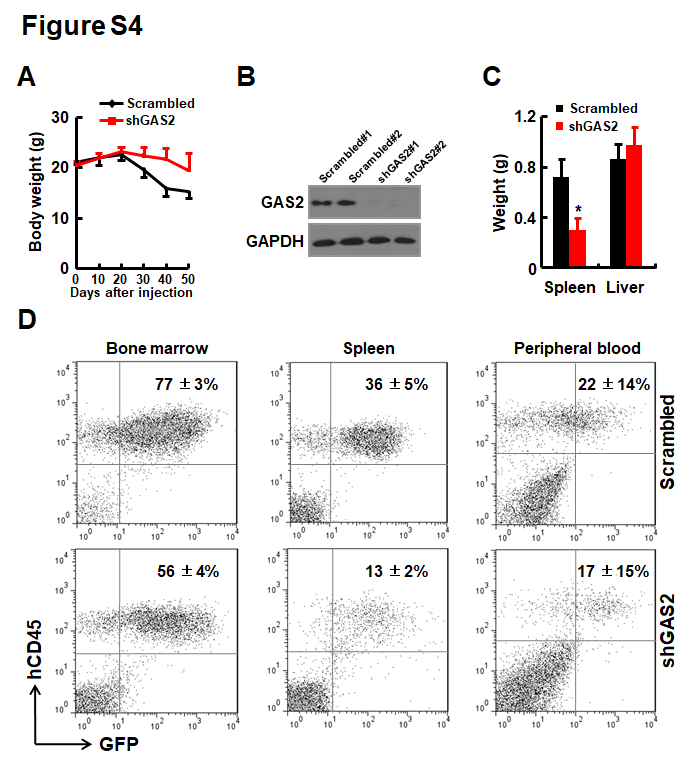
**

**Supplementary Fig. S4.** GAS2 silencing inhibits Jurkat cells to generate leukemia in immunodeficient mice. (A) The mice body weights of the control (scrambled) and GAS2 silenced (shGAS2) groups were monitored through day 1 to 50 post tail vein injection. (B) The leukemic cells (hCD45^+^GFP^+^) were collected from the diseased mice, and Western blot was performed to analyze the expression of GAS2. (C) The weights of spleen and liver in Scrambled and shGAS2 groups were compared. (D) Flow cytometry was conducted to analyze the leukemic cells in the bone marrow, spleen, and peripheral blood of the mice from both groups. Data were represented as the mean ± SEM, and the statistical significance was estimated with Student’s *t*-test. (*, *P*<0.05.)

**
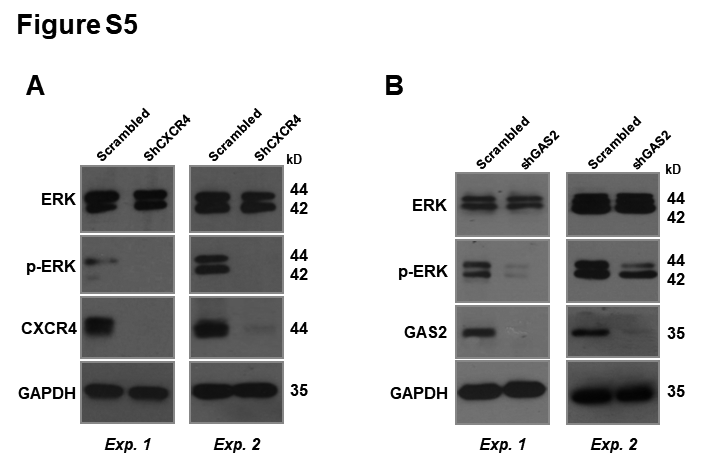
**

**Supplementary Fig. S5.** CXCR4 or GAS2 silencing inhibits ERK phosphorylation. Western blots were conducted to analyze the expression of ERK and p-ERK in Jurkat cells upon CXCR4 or GAS2 silencing.


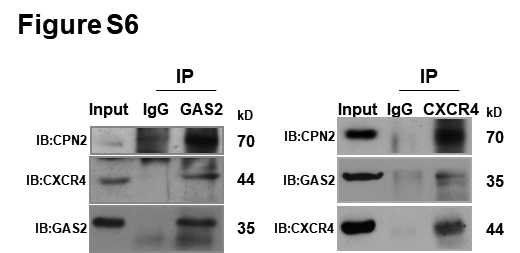


**Supplementary Fig. S6.** Interaction between GAS2 and CXCR4 in Jurkat cells. Immunoprecipitation (IP) against GAS2 in Jurkat cells was performed (left panel), and the expression of CXCR4 and Calpain2 (CPN2) was detected with immunoblot (IB). Conversely, IP against CXCR4 in Jurkat cells was conducted (right panel), and the expression of GAS2 and CPN2 was detected with IB.


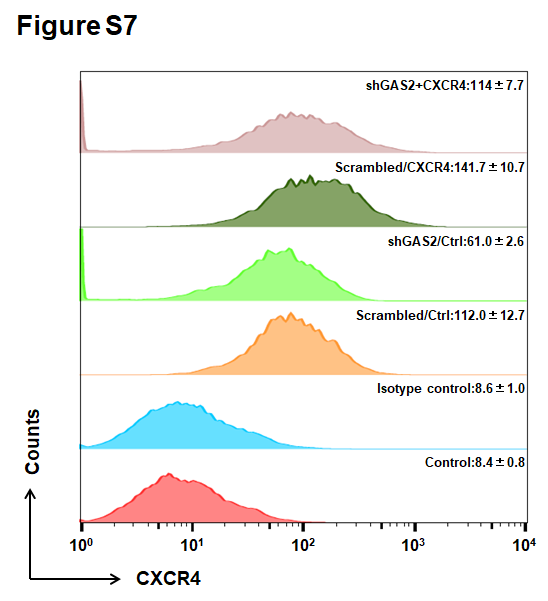


**Supplementary Fig. S7.** The surface expression of CXCR4 in Jurkat cells upon various viral infections. Jurkat cells were transduced with a combination of various lentiviruses, as indicated. The surface expression of CXCR4 of these cells were then analyzed by flow cytometry (n=3). Data were represented as the mean ± SEM.

**
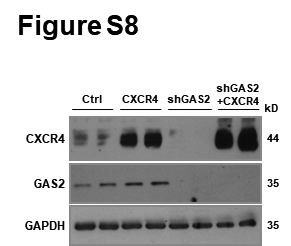
**

**Supplementary Fig. S8.** The expression of GAS2 and CXCR4 in leukemic cells were detected. Variously transduced Jurkat cells were injected into immunodeficient mice through tail vein, including the control (Ctrl), CXCR4, shGAS2, and shGAS2+CXCR4 cells. The leukemic cells from each group of mice were subjected to Western blot to analyze the expression of GAS2 and CXCR4.

**
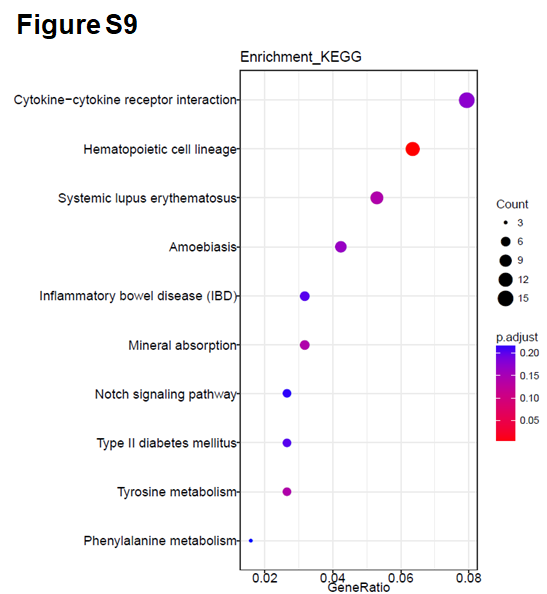
**

**Supplementary Fig. S9.** [Kyoto Encyclopedia of Genes and Genomes](https://www.baidu.com/link?url=-IVQbAAfcb06XBUSSf_YloKwrOH_5LCwzRrH5bPVD6u&wd=&eqid=c740263a0009c67e00000003606958e7) (KEGG) enrichment analysis of differentially expressed transcripts comparing GAS2 silenced Jurkat cells with their control.


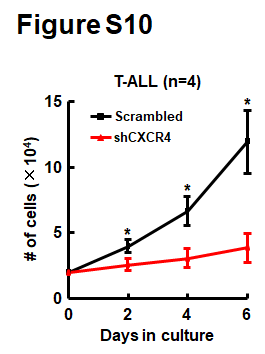


**Supplementary Fig. S10.** CXCR4 silencing inhibits the growth of primary T-ALL cells. The bone marrow cells from T-ALL patients (n=4) were activated with CD3/CD28, and cultured with a T cell expansion medium supplemented with IL-2 for two days. These cells were transduced with lentiviral vectors for the delivery of the control (Scrambled) and shRNA against CXCR4 (shCXCR4). Three days later, GFP^+^ cells were isolated by FACS, and their growth analyzed. Data were represented as the mean ± SEM, and the statistical significance was estimated with Student’s *t*-test. (*, *P*<0.05)


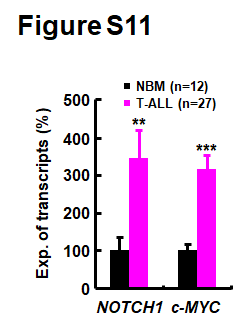


**Supplementary Fig. S11.** NOTCH1 and c-MYC are aberrantly expressed in T-ALL patients. The T-ALL cells from patients (n=27) and CD3^+^ cells from normal bone marrow (NBM) of healthy donors (n=12) were used for RNA preparation, and the expression of *NOTCH1* and *c-MYC* was assessed by RT-qPCR. Data were represented as the mean ± SEM, and the statistical significance was estimated with Student’s *t*-test. (**, *P*<0.01 and ***, *P*<0.001)


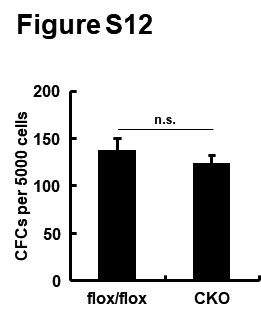


**Supplementary Fig. S12.** Colony-forming cell production of the bone marrow cells from CKO and flox/flox mice. The bone marrow cells from CKO mice (n=4) and flox/flox mice (n=4) were harvested and plated for colony-forming cell (CFC) assays. Data were represented as the mean ± SEM, and the statistical significance was estimated with Student’s *t*-test. n.s., not significant.


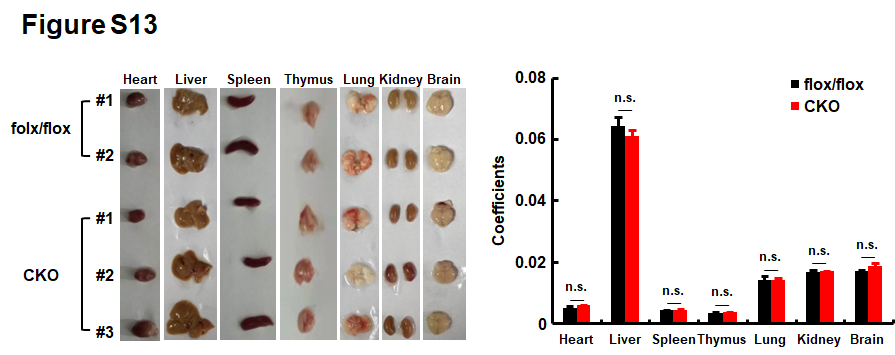


**Supplementary Fig. S13.** Gas2 depletion has no evident effects on the coefficients of major organs. The representative photos of major organs including heart, liver, spleen, thymus, lung, kidney, and brain from both flox/flox and flox/flox;Vav-iCre (conditional knockout, CKO) groups of mice were shown, and the coefficients (ratio of organ weight to body weight) of these organs were compared (n=8). Data were represented as the mean ± SEM, and the statistical significance was estimated with Student’s *t*-test. n.s., not significant.
